# A revision of the Nearctic species of *Liancalus* Loew (Diptera, Dolichopodidae)

**DOI:** 10.3897/zookeys.483.9222

**Published:** 2015-02-23

**Authors:** Justin B. Runyon, Richard L. Hurley

**Affiliations:** 1 Rocky Mountain Research Station, USDA Forest Service, 1648 S. 7th Avenue, Bozeman, Montana 59717, USA; 2 Montana Entomology Collection, Montana State University, Room 50 Marsh Laboratory, Bozeman, Montana 59717, USA; 3 Deceased, formerly, Montana Entomology Collection, Montana State University, Room 50 Marsh Laboratory, Bozeman, Montana 59717, USA

**Keywords:** Nearctic, long-legged flies, Dolichopodidae, Hydrophorinae, wings, new species, courtship display

## Abstract

The genus *Liancalus* Loew is revised for the Nearctic Region. Seven species are documented from this region including two new species: *Liancalus genualis* Loew, *Liancalus hydrophilus* Aldrich, *Liancalus limbatus* Van Duzee, *Liancalus pterodactyl* **sp. n.**, *Liancalus querulus* Osten Sacken, *Liancalus similis* Aldrich, and *Liancalus sonorus* **sp. n.** Lectotypes are designated for the following species: *Liancalus genualis*, *Liancalus hydrophilus*, *Liancalus querulus*, and *Liancalus similis*. The species are illustrated, a key to males and females is provided, and their distributions mapped. Adults of *Liancalus* are some of the largest species of Dolichopodidae and commonly occur on waterfalls and vertical seeps.

## Introduction

**Figure 1.**
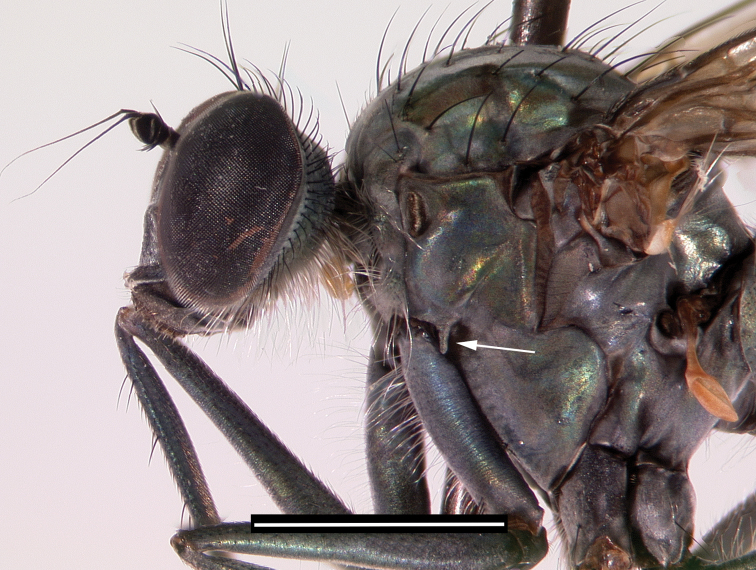
Lateral view of head and thorax of the holotype of *Liancalus pterodactyl* sp. n. The finger-like projection on the proepimeron is indicated with an arrow; this lobe is diagnostic for the genus *Liancalus*. Scale bar = 1 mm.

*Liancalus* Loew, is a genus of long-legged flies in the subfamily Hydrophorinae containing 21 described species from all zoogeographical regions except Australasia and Oceania. Six species are known from the Palearctic Region, seven from the Nearctic Region, five from the Afrotropical Region, and three from the Oriental Region (Yang et al. 2006). Adults have a diagnostic finger-like projection on the proepimeron near base of coxa I (Fig. 1). *Liancalus* includes some of the largest species of Dolichopodidae (body length approaches 12 mm in some species) and are commonly found in madicolous habitats, especially on or near waterfalls and seeps.

**Figure 2.**
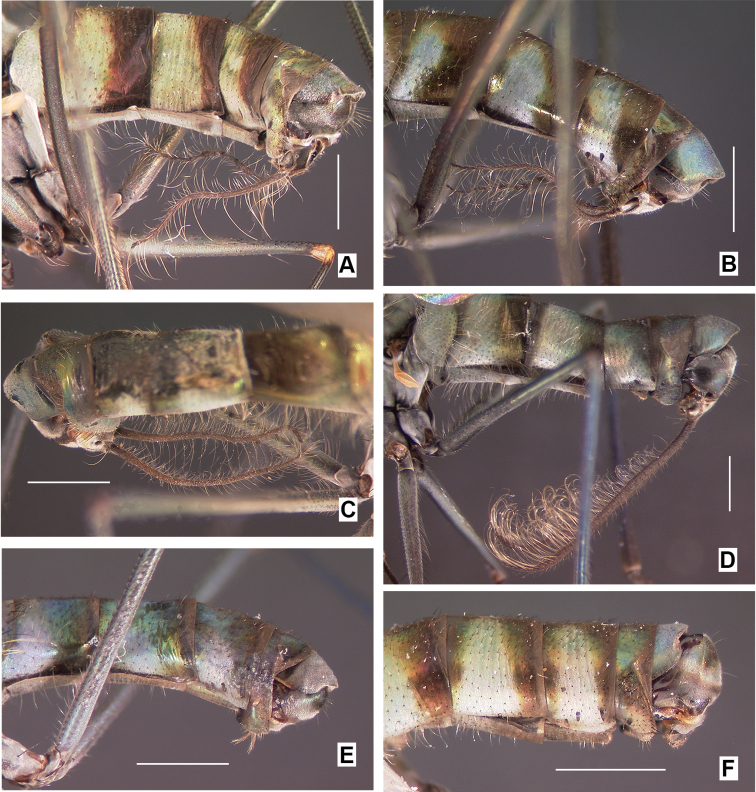
Lateral view (unless otherwise noted) of abdomens and postabdomens of males, **A** *Liancalus genualis* Loew **B** *Liancalus sonorus* sp. n. **C** *Liancalus limbatus* Van Duzee (dorsolateral view) **D** *Liancalus pterodactyl* sp. n. **E** *Liancalus similis* Aldrich, and **F** *Liancalus querulus* Osten Sacken. Scale bars = 1 mm. See Figure 8 for *Liancalus hydrophilus*.

**Figure 3.**
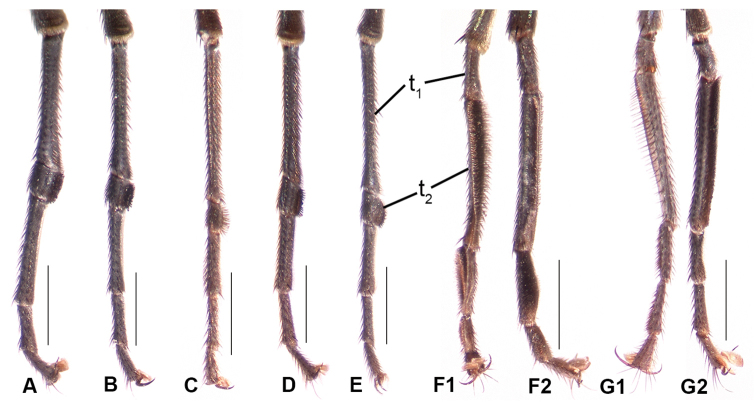
Front tarsi of males, **A** *Liancalus pterodactyl* sp. n., medial view **B** *Liancalus hydrophilus* Aldrich, medial view **C** *Liancalus genualis* Loew, medial view **D** *Liancalus limbatus* Van Duzee, medial view **E** *Liancalus sonorus* sp. n., medial view **F1** *Liancalus querulus* Osten Sacken, ventral view **F2** *Liancalus querulus*, medial view **G1** *Liancalus similis* Aldrich, ventral view **G2** *Liancalus similis*, medial view. Scale bars = 0.5 mm. t_1_ = tarsomere 1, t_2_ = tarsomere 2.

**Figure 4.**
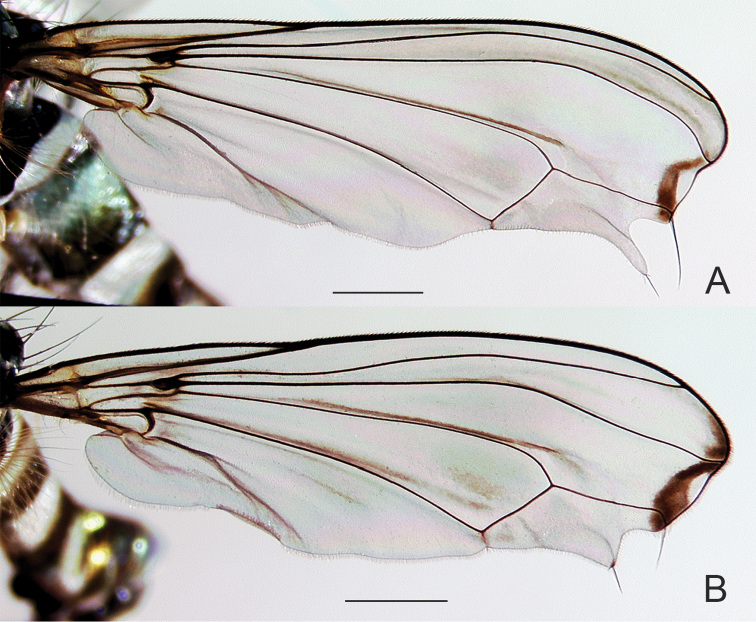
Wings of male **A** *Liancalus pterodactyl* sp. n., and **B** *Liancalus hydrophilus* Aldrich. Scale bars = 1 mm.

**Figure 5.**
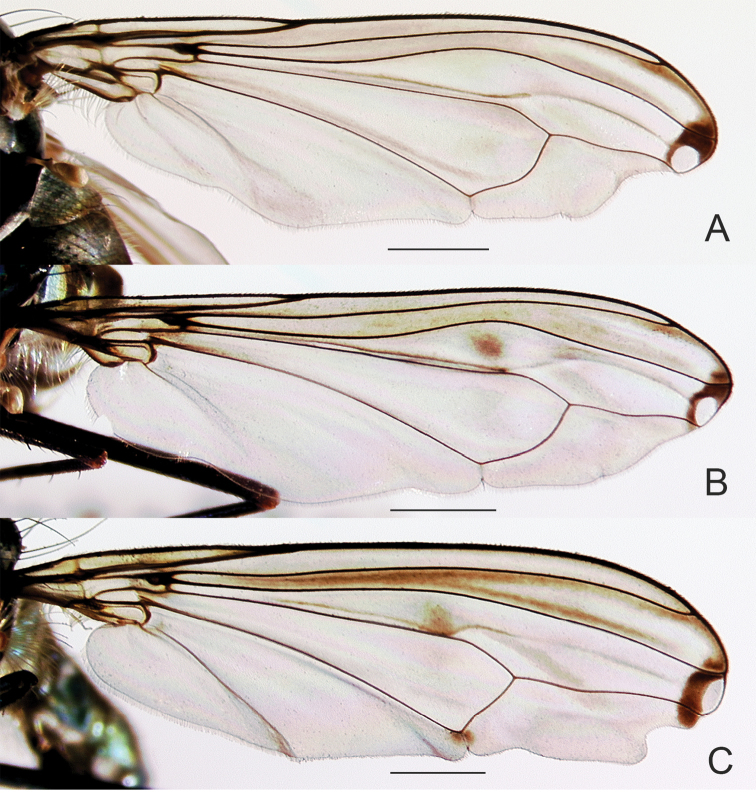
Wings of male **A** *Liancalus genualis* Loew **B** *Liancalus sonorus* sp. n., and **C** *Liancalus limbatus* Van Duzee. Scale bars = 1 mm.

**Figure 6.**
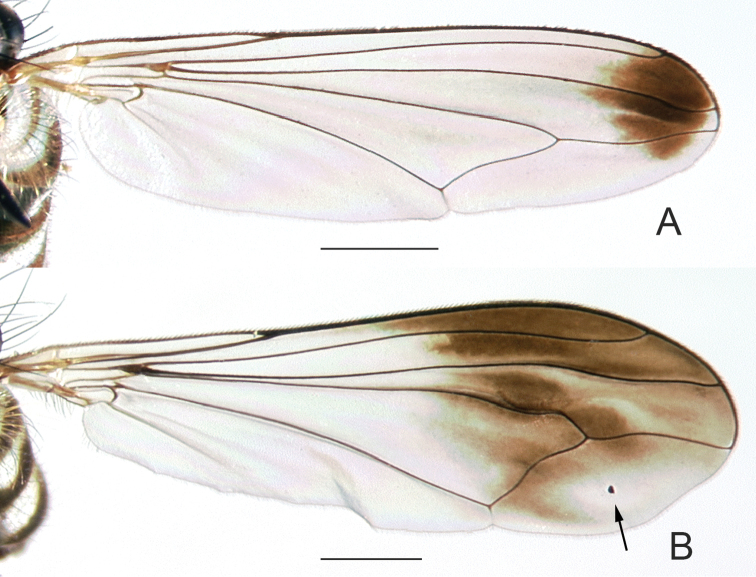
Wings of male **A** *Liancalus querulus* Osten Sacken, and **B** *Liancalus similis* Aldrich. Scale bars = 1 mm. Note black speck in cell m of *Liancalus similis*, indicated by arrow.

**Figure 7.**
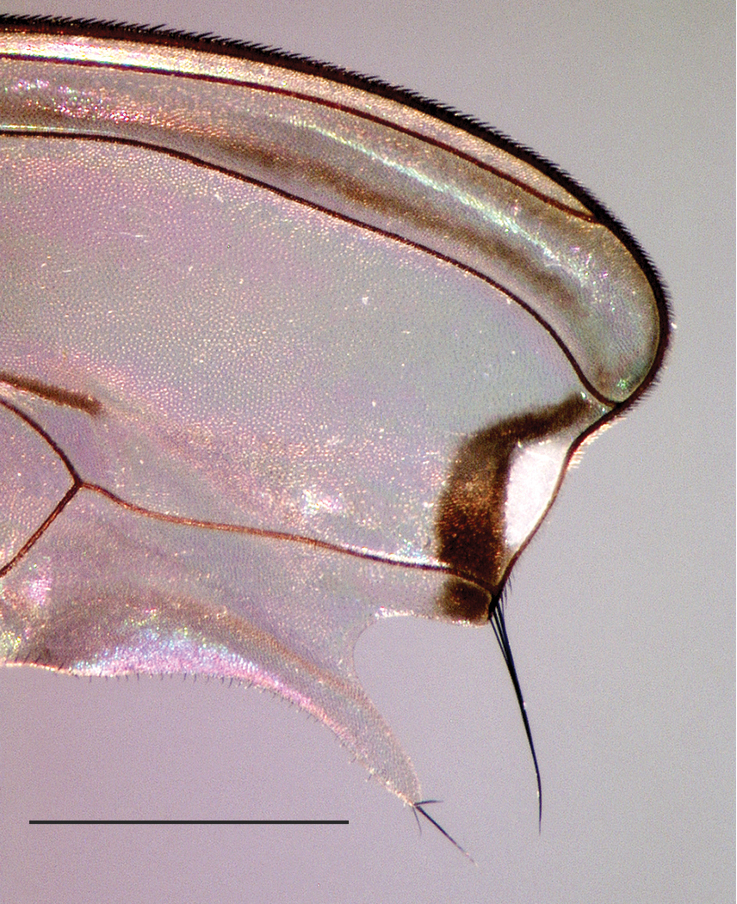
Wing tip of male *Liancalus pterodactyl* sp. n., showing white reflectance of apical spot in certain lights. Scale bar = 1 mm.

Males are readily identified to species using secondary sexual structures such as length of the cerci (Fig. 2), relative length of the first two segments of the fore tarsi (Fig. 3), and most easily, by their modified wings (Figs 4–6). The modified wings of males likely serve a signaling function in courtship. Males of several species have been observed standing in front of females and fanning their wings, e.g., the Palearctic *Liancalus virens* (Scopoli) (Crossley 1988). We have also observed similar behavior in *Liancalus pterodactyl* sp. n. and *Liancalus similis* Aldrich). The wing tips of males of many *Liancalus* species, including all seven Nearctic species, have darkened areas often enclosing a white spot (Figs 4–5, 7), which would increase the signal contrast during fanning (see Fig. 2 in Zimmer et al. 2003). Males of some species also have lobes and long hairs near their wing tips (Figs 4, 7) that could signal females via sounds produced during fanning, similar to what has been proposed for species of *Erebomyia* Runyon & Hurley (Runyon and Hurley 2004, Hurley and Runyon 2009). The modified fore tarsi in males may also play a role in courtship (see Remarks for *Liancalus limbatus* Van Duzee).

Like most Dolichopodidae, little is known about their biology. Adults and larvae are predators and adults have been documented feeding on arthropods, especially larvae of nematocerous Diptera (Masunaga 2001, Ulrich 2004). Adult females of *Liancalus similis* have been observed feeding on nematocerous larvae in Montana (Suppl. material 1). Larvae of *Liancalus similis* were found within algal mats below waterfalls and described by Corpus (1986); larvae of the Palearctic *Liancalus virens* (Vaillant 1948) and the Oriental *Liancalus zhenzhuristi* (Masunaga 2001) have also been described. Mouthparts of adults (Cregan 1941) and larvae (Sinclair 1992) have been described and illustrated for several Nearctic species.

In 1945, Harmston and Knowlton stated, "Possessing long, graceful bodies, delicately tinted with brilliant hues of bronze and purple against a green background, few Diptera are more beautiful than the species of *Liancalus*". Here, we revise these beautiful flies occurring in the Nearctic, illustrate the species, map their distributions, and provide a key to species.

### Notes on nomenclature

Loew (1857: 22) originally used the name *Liancalus* stating: "Will man diese Gruppe zu einer Gattung erheben, so ist der mir von Herrn Haliday brieflich vorgeschlagene Name *Liancalus* ein ganz passender. Herr Rondani hat den bereits früher an eine Käfergattung vergebenen Namen *Anoplomerus* dafür vorgeschlagen, der verworfen werden muss". ["If one wants to raise this group to a genus, then the name *Liancalus* suggested by Mr. Haliday in a letter is completely suitable. Mr. Rondani has already proposed the name *Anoplomerus* for this, which has previously been given to a beetle genus and which must be rejected"]. Most subsequent workers have interpreted Loew’s use of the name *Liancalus* to be a replacement name for *Anoplomerus* (e.g., Pollet et al. 2004; Yang et al. 2006), but Coquillett (1910) obviously considered it a new genus-group when he designated a type species. Loew clearly stated that this group already had a name – *Anoplomerus* – but that this name had to be rejected and that *Liancalus* is a suitable name for the group. This would indicate that Loew intended *Liancalus* as a replacement name.

Following this logic, i.e. *Liancalus* is a replacement name for *Anoplomerus*, would cause significant nomenclatural instability and confusion. This is because Rondani (1856), in the unpaginated last page of his work, changed the type species of *Anoplomerus* from "*Hydrophorus Regius* Fabr." [now treated as a *Liancalus* Loew, 1857] to "*Hydrophorus Notatus* Meig." [now treated as a *Scellus* Loew, 1857] (discussed in Hurley 1995 and O’Hara et al. 2011). As a replacement name, *Liancalus* Loew would automatically take the type species of the name replaced, *Hydrophorus notatus* Meigen, making *Liancalus* a synonym of *Scellus*, and leaving the species currently in *Liancalus* without a generic name, thus threatening current usage of both *Liancalus* and *Scellus*, names and concepts that have been frequently and widely used. Therefore, an application has been submitted to the International Commission on Zoological Nomenclature (Runyon et al. submitted) to invoke its Plenary Powers under Article 78 (ICZN 1999) to conserve both generic concepts as presently used. Under Article 82.1, we are therefore required to maintain prevailing usage.

## Materials and methods

This study was made possible by examination of more than two thousand specimens from the following collections, listed below with codens in parentheses; codens follow "The Insect and Spider Collections of the World" website (Evenhuis 2014): American Museum of Natural History, New York, USA (AMNH); Brigham Young University, Provo, USA (BYU); California Academy of Sciences, San Francisco, USA (CAS); Carnegie Museum of Natural History, Pittsburgh, USA (CMNH); Canadian National Collection of Insects, Ottawa, Canada (CNC); California State Collection of Arthropods, Sacramento, USA (CSCA); Colorado State University, Ft. Collins, USA (CSUC); Cornell University Insect Collection, Ithaca, USA (CUIC); University of Guelph Insect Collection, Guelph, Canada (DEBU); Essig Museum of Entomology, University of California, Berkeley, USA (EMEC); Utah State University Insect Collection, Logan, USA (EMUS); Florida State Collection of Arthropods, Gainesville, USA (FSCA); Illinois Natural History Survey, Champaign, USA (INHS); Iowa State University Insect Collection, Ames, USA (ISUI); Los Angeles County Museum of Natural History, Los Angeles, USA (LACM); Museum of Comparative Zoology, Harvard University, Cambridge, USA (MCZ); Michigan State University, East Lansing, USA (MSUC); Montana Entomology Collection, Bozeman, USA (MTEC); New York State Museum, Albany, USA (NYSM); Oregon Department of Agriculture Insect Museum, Salem, USA (ODAC); Oregon State Arthropod Collection, Corvallis, USA (OSAC); Ohio State University, Columbus, USA (OSU); Snow Entomological Collections, University of Kansas, Lawrence, USA (SEMC); University of Arizona Insect Collection, Tucson, USA (UAIC); Spencer Entomological Museum, University of British Columbia, Vancouver, Canada (UBCZ); R.M. Bohart Museum of Entomology, University of California, Davis, USA (UCD); University of California, Riverside, USA (UCR); University of Georgia Collection of Arthropods, Athens, USA (UGCA); University of Minnesota, St. Paul, USA (UMSP); United States National Museum of Natural History, Washington, District of Columbia, USA (USNM); W.F. Barr Entomological Collection, Pullman, USA (WSU); University of Idaho, Moscow, USA (WFBM); University of Michigan, Ann Arbor, USA (UMMZ); James Entomological Collection, Washington State University, Pullman, USA (WSU).

Descriptions of structural terminology follow McAlpine (1981), except for genitalia which follow Sinclair and Cumming (2006). Illustrations of male genitalia are shown approximately as they appear on intact specimens (rotated approximately 180° and lateroﬂexed to the right), but in descriptions "dorsal" and "ventral" refer to the morphological positions before rotation and lateroﬂexion (e.g., top of page in Figs 10–12 is ventral), with the top of the page ventral and the bottom of the page dorsal. Genitalia were cleared first using 10% KOH and then 85% lactic acid then mounted in glycerin for examination and illustration. Body length was measured from the base of antenna to tip of the abdomen. Wing length was measured from the humeral crossvein to the wing apex. Density of pollen is characterized as in Runyon and Hurley (2003), Runyon (2008), and Hurley and Runyon (2009).

The following abbreviations and terms are used: ad = anterodorsal(ly); av = anteroventral(ly); pd = posterodorsal(ly); pv = posteroventral(ly); T1, T2, etc. = abdominal tergite one, abdominal tergite two, etc.; S1, S2, etc. = abdominal sternite one, abdominal sternite two, etc. Legs are designated by roman numerals, tarsomeres by bracketed Arabic numerals (e.g., Tarsus III(4) = 4th tarsomere of hindleg).

Label data for type specimens are cited verbatim in quotation marks. Lines on a label are separated by a slash (/); labels on a pin are separated by semicolons; additional information is included in square brackets ([ ]). The repository of each type is given in parentheses. Label data for other specimens are summarized using a standardized format. For U.S. states, each county is only given once followed by localities from that county which are separated by commas; counties are separated by semicolons. The following abbreviations are used in the ‘‘material examined’’ sections: Crk – Creek; Co – County; Cpgd – Campground; Cyn – Canyon; E – east; Hwy – Highway; mi. – miles; Mt/Mts – Mountain/Mountains; N – north; NF – National Forest; NP – National Park; nr – near; PK – Park; PP – Provincial Park; R – River; S – south; SP – State Park; Spr/Sprs – spring/springs; Tr – Trail; W – west. The following abbreviations for frequent collectors are used: ALM – A.L. Melander; DDW – D.D. Wilder; PHA – P.H. Arnaud Jr.; JBR – J.B. Runyon; RLH – R.L. Hurley; WJT – W.J. Turner; FCH – F.C. Harmston; JRV – J.R Vockeroth; JMA – J. M. Aldrich; KJG – K.J. Goeden.

## Taxonomy

### Genus Liancalus

Taxon classificationAnimaliaDipteraDolichopodidae

Loew, 1857

Anoplomerus Rondani, 1856: Rondani 1856: 141. Type species: *Dolichopus regius* Fabricius, 1805 treated as type species, awaiting ruling by the International Commission on Zoological Nomenclature. Preoccupied by *Anoplomerus* Guérin-Méneville, 1844.
Anoplopus Rondani, 1857: Rondani 1857: 14. Replacement name for *Anoplomerus*Rondani 1856 [Not Guérin-Méneville 1844]. Type species taken as that of replaced name under ICZN Art. 67.8: *Dolichopus regius* Fabricius (pending ICZN ruling). Preoccupied by *Anoplopus* Wagler, 1830.
Liancalus Loew, 1857: Loew 1857: 22. Replacement name for *Anoplomerus* Rondani, 1856 [Not Guérin-Méneville 1844]. Type species taken as that of replaced name under ICZN Art. 67.8: *Dolichopus regius* Fabricius. Bigot 1859: 230; Loew 1861: 69–70; Loew 1864: 198–200; Osten Sacken 1877: 318; Gobert 1887: 33; Bigot 1890: 277; Aldrich 1893: 569; Becker et al. 1903: 343–344; Aldrich 1904: 271; Aldrich 1905: 298; Coquillett 1910: 561; Lundbeck 1912: 22, 352–356; Wahlgren 1912: 5, 48; Frey 1915: 74; Van Duzee 1917: 126; Becker 1917–1918: 160, 193; Becker 1922a: 117–119; Becker 1922b: 41; Curran 1926: 406–407; Parent 1932: 121–122; Curran 1934: 217; Parent 1938: 19, 268, 306; Parent 1939: 276; Harmston and Knowlton 1945: 55–56; Robinson 1964: 118, 182; Dyte 1967: 123; Cole 1969: 272, 282; Robinson 1970a: 59, 62; Robinson 1970b: 57; Dyte 1975: 241–242; D’Assis Fonseca 1978: 41; Negrobov 1978: 416–417; Negrobov 1979: 928; Robinson and Vockeroth 1981: 633, 635, 637; Hurley 1985: 3; Negrobov et al. 1987: 157–158; Negrobov 1991: 41; Wei and Liu 1995: 35; Yang 1998: 153; Masunaga 2001: 109, 117–118; Pollet et al. 2004: 52; Yang et al. 2006: 19, 246; Bickel 2009: 683; Evenhuis and Bickel 2011: 4–5; O’Hara et al. 2011: 30; Yang et al. 2011: 363; Kahanpää 2014: 203.

#### Notes.

The type species of *Liancalus* is involved in a convoluted nomenclatural issue resulting from an unpaginated correction page at the end of Rondani (1856) and interpretation of Loew’s (1857) creation of the name. If a straightforward use of the ICZN rules were to be followed, the genus here and previously considered *Liancalus* Loew would lack a valid generic name. However, the purpose of the ICZN is to "Promote stability and universality" (ICZN Preamble), and when strict application of the Code would act contrary to this purpose, the International Commission on Zoological Commission is empowered to set aside the rules using their Plenary Powers (ICZN Art. 78.1). Therefore, a petition has been prepared for the ICZN (Runyon et al. submitted) asking that they use their Plenary Powers to preserve prevailing usage of *Liancalus* by setting aside the type species under the Rules, and replace it with a type species that retains established and universal usage. In the meantime, under ICZN Art. 82.1, prevailing use is to be maintained until such time as the Commission’s ruling is published. Therefore, *Liancalus* will be treated herein as if its type species is *Dolichopus regius* Fabricius, 1805.

#### Diagnosis.

**Figure 8.**
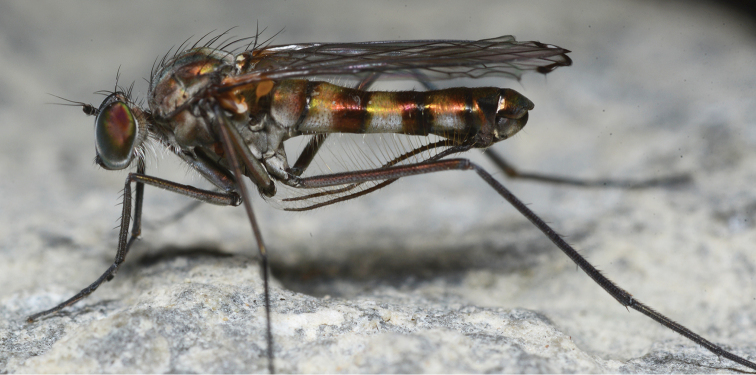
Habitus photograph of male *Liancalus hydrophilus* Aldrich taken at a waterfall in Utah County, Utah, N39.95963°, W111.2678°, on 13 September 2013. Note long cerci with nearly uniform row of long yellow setae. Photo taken by C. Riley Nelson.

**Figure 9.**
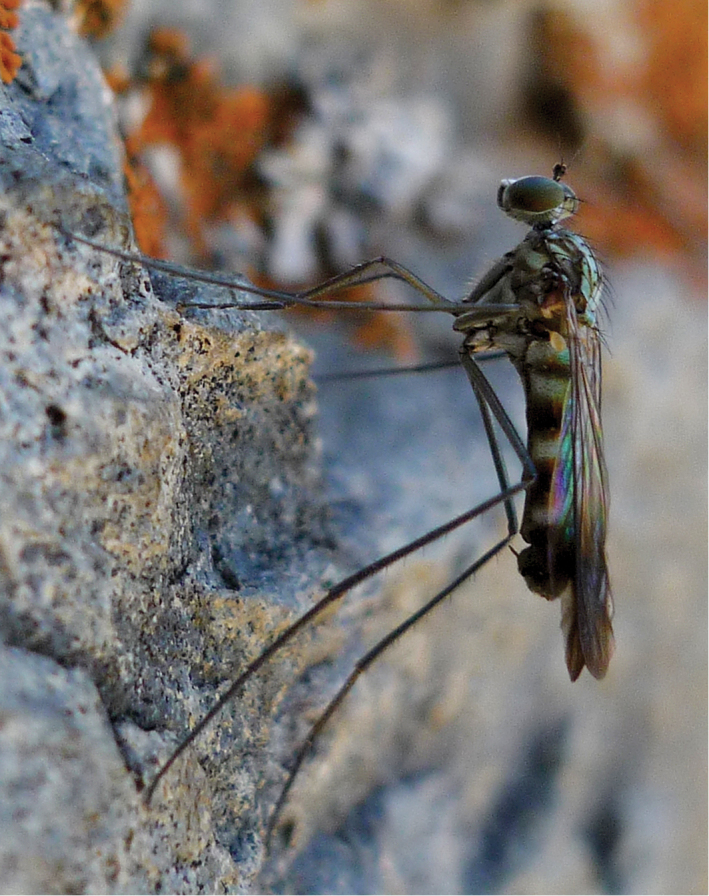
Habitus photograph of male *Liancalus similis* Aldrich taken at Palisade Falls in Gallatin County, Montana on 12 October 2012. Note very short cerci. Photo taken by Justin Runyon.

Large flies of rather uniform general color and appearance (Figs 8–9) whose males and females can be recognized by the finger-like projection ventrally from proepimeron near base of coxa I (Fig. 1).

#### Description.

**Male.** Body length 6.5–12.0 mm, wing length 6.0–8.5 mm.

**Head:** Face and frons broadly separated with distinct frontoclypeal suture near mid-face (Fig. 1). Eyes with short hairs between facets. Vertical setae on small elevation; ocellar tubercle prominent with 2 large setae, without hairs; with 2 postocellar setae. Gena absent. Proboscis somewhat sclerotized, slightly enlarged, covered with sparse gray-brown pollen; each labellar lobe with 6 geminately sclerotized pseudotracheae. Antenna of rather uniform shape, size, and color (Figs 1, 8–9); black, scape without dorsal setae; pedicel with apical ring of setae/setulae, longest setae dorsally and ventrally; first flagellomere about as long as wide, broadly pointed apically, arista inserted near midpoint of dorsal edge.

**Thorax:** Scutum metallic green to green-blue with silver-gray pollen and bronze-red stripes; 0–14 acrostichal setae in a single row; usually 6 dorsocentral setae (6–10 in *Liancalus pterodactyl*), 2 notopleural setae; 1–3 strong, black postpronotal setae (often with some smaller white hairs), usually 2 presutural intra-alar setae (1 in *Liancalus genualis*), 1 presutural seta, 2 postsutural supra-alar setae, and 1 postalar seta per side; scutellum usually with 6 large marginal setae (8–9 in *Liancalus pterodactyl*), no additional hairs; proepisternum with 1 dorsal and 1 ventral tuft of white hairs. Pleura metallic bronze-green, covered with dense silver-gray pollen, without setae or hairs (Fig. 1).

**Legs:** Legs very long, slender, dark metallic green (Figs 8–9). Coxa I uniformly covered with white hairs on anterior surface; coxa II with white hairs on anterior surface and black *ad* seta near middle; coxa III with a black dorsal seta near middle (Fig. 1). Femur II and III with a slender preapical *ad* seta near 3/4. Tarsus I either with tarsomere 1 long and tarsomere 2 short (Figs 3A–E), or with tarsomere 1 short and tarsomere 2 long (Figs 3F–G).

**Wing:** Modified with dark brown markings and spots, sometimes enclosing a white apical spot and sometimes with lobes and setae (Figs 4–7). Calypter yellow with a fan of long, pale yellow setae. Halter pale yellow.

**Figure 10.**
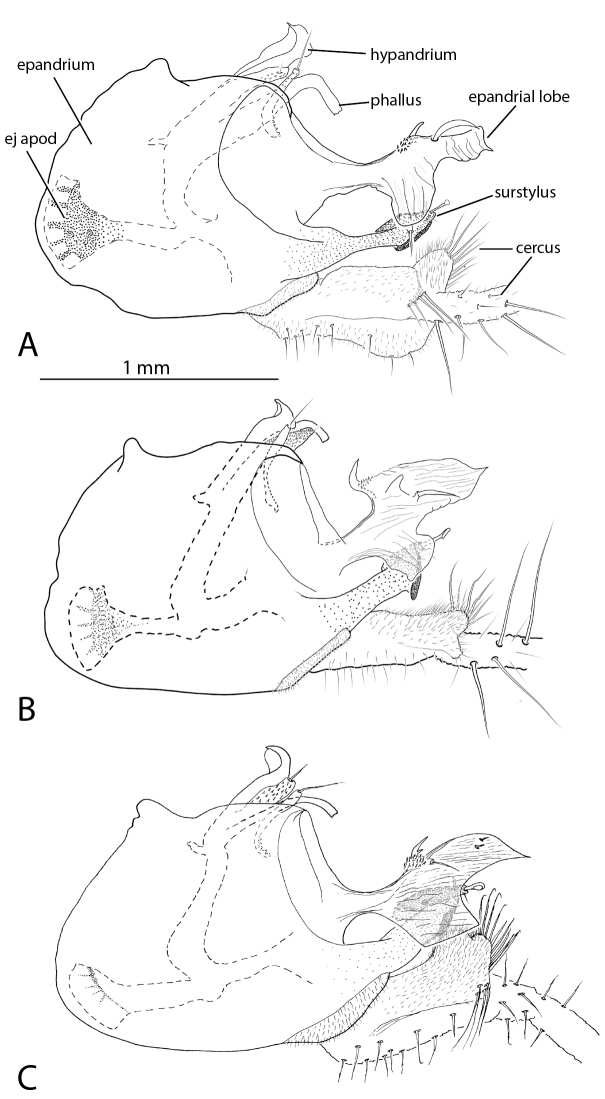
*Liancalus* male terminalia, right lateral view of **A** *Liancalus genualis* Loew **B** *Liancalus sonorus* sp. n., and **C** *Liancalus limbatus* Van Duzee. ej apod = ejaculatory apodeme. Scale bar = 1 mm. Only base of cercus shown, see Fig. 2 for photographs of entire cerci.

**Figure 11.**
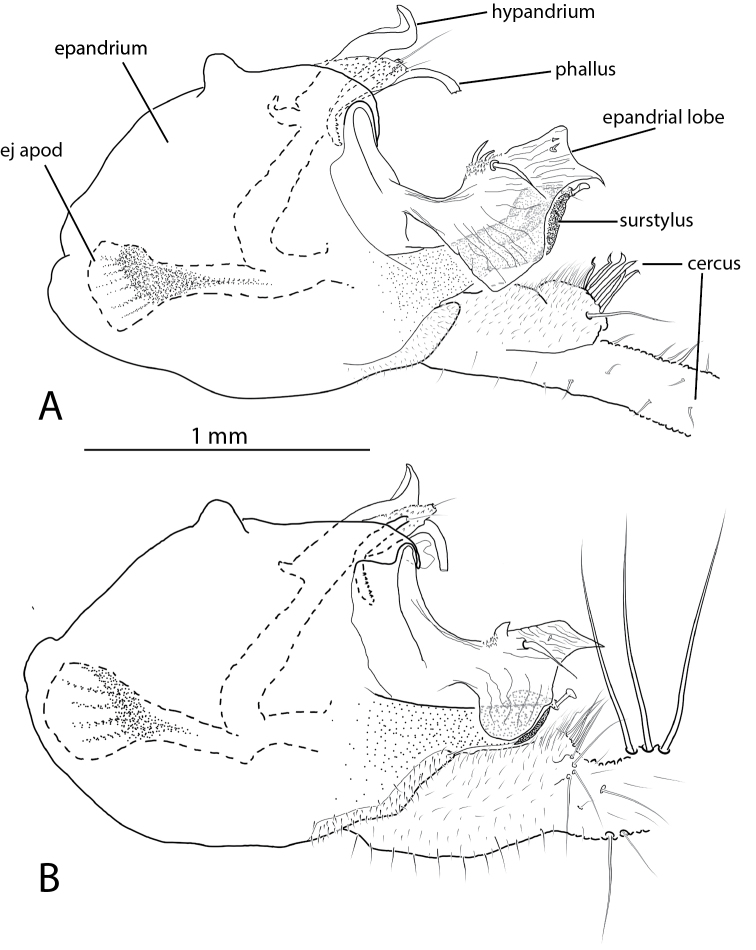
*Liancalus* male terminalia, right lateral view of **A** *Liancalus pterodactyl* sp. n., and **B** *Liancalus hydrophilus* Aldrich. ej apod = ejaculatory apodeme. Scale bar = 1 mm. Only base of cercus shown, see Figures 2D and 8 for photographs of entire cerci.

**Figure 12.**
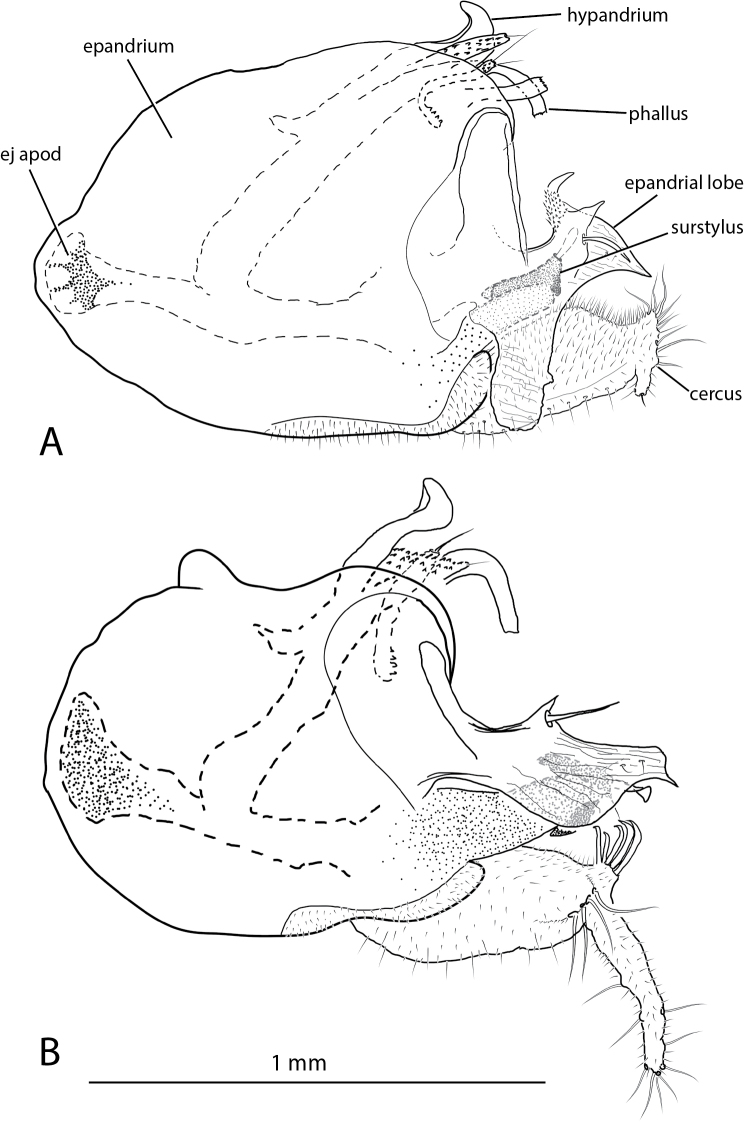
*Liancalus* male terminalia, right lateral view of **A** *Liancalus querulus* Osten Sacken, and **B** *Liancalus similis* Aldrich. ej apod = ejaculatory apodeme. Scale bar = 1 mm.

**Abdomen:** Cylindrical, elongate, and slightly broadened at apex (Fig. 2). T5 prolonged ventrally into two lateral flap-like projections that form a hood or pocket for the apex of the hypopygium. Hypopygium (Figs 10–12) nearly round, capping apex of abdomen. Phallus arched to rather sharply bent dorsally just before apex, with apical margin minutely serrate. Hypandrium rather broad, thin, arched anteriorly near apex with lateral lobe bearing setulae and a larger seta at or near apex. Epandrium with large, apical, thin, nearly transparent lobe that is hinged and can be raised or lowered dorsoventrally; at rest, this lobe sits against and covers the surstylus and base of cerci. Surstylus somewhat pointed, strongly sclerotized, directed medially, with large spatulate seta near apex. Cerci broad basally, with either very short (Figs 2E–F, 12) or very long filaments that project anteriorly below abdomen (Figs 2A–D, 8).

**Female.** Body length 5.0–9.0 mm, wing length 5.5–7.5 mm. Lacking typical male secondary sexual characters and similar to male except: face broader, nearly parallel-sided; palpi larger; fore tarsi unmodified; wings unmodified, but with diagnostic dark brown spots in most specimens (Fig. 16); abdomen shorter and somewhat flattened dorsoventrally.

**Immatures.** Larvae twelve segmented, cylindrical, truncate posteriorly and tapered anteriorly, opalescent with transparent cuticle; antenna with basal ring bearing sensilla; mouthparts dark brown to black, labrum large with pointed tip (sometimes hooked and/or with tooth-like projections), mandibular hook well developed; metacephalic rods enlarged at caudal tips, longer than tentorial arm. Pupa with prothoracic respiratory horns about 2 mm long, sharply pointed at tips; frontofacial sutures distinct, brown; abdominal segments 2–7 with rows of posteriorly-directed spines. Coccon elliptical, externally composed of sand grains and sometimes moss and mud, inner surface smooth; respiratory horn tips exposed. See Vaillant (1948), Corpus (1986) and Masunaga (2001) for illustrations and photographs of immature stages.

#### Remarks.

Cerci of male specimens sometimes shrivel upon drying. In teneral specimens, the spots on the wings can be very faint which can render them, particularly females, difficult to identify.

### Key to Nearctic species of *Liancalus* Loew

**Table:** 

1	Male	**2**
–	Female	**8**
2	Cerci very short (Fig. 2E–F); foretarsus with tarsomere 1 short and tarsomere 2 long (Fig. 3F–G)	**3**
–	Cerci very long (Figs 2A–D, 8); foretarsus with tarsomere 1 long and tarsomere 2 very short (Fig. 3A–E)	**4**
3	Wing with brown clouding on most of apical half and a small black speck near center of cell m (Fig. 6B)	***similis* Aldrich**
–	Wing with semicircular brown cloud near apex, without black speck in cell m (Fig. 6A)	***querulus* Osten Sacken**
4	Wing with outstanding setae on posteroapical margin (Figs 4, 7)	**5**
–	Wing without obvious setae on posteroapical margin (Fig. 5)	**6**
5	Wing margin with proximal setae arising from apex of long, slender, finger-like lobe (Figs 4A, 7); cerci with long setae confined to apical half (Fig. 2D)	***pterodactyl* sp. n.**
–	Wing margin with proximal setae arising from short, rounded lobe (Fig. 4B); cerci with long, pale setae along full-length (Fig. 8)	***hydrophilus* Aldrich**
6	Crossvein dm-cu nearly straight (Fig. 5C)	***limbatus* Van Duzee**
–	Crossvein dm-cu strongly arched (Fig. 5A–B)	**7**
7	Wing margin excavated posterior to vein M_1_ (Fig. 5A); intra-alar seta at transverse suture missing (eastern North America)	***genualis* Loew**
–	Wing margin without or with very small excavation posterior to vein M_1_ (Fig. 5B); intra-alar seta at transverse suture present (Arizona, Mexico)	***sonorus* sp. n.**
8	Intra-alar seta at transverse suture absent; wing as in Fig. 16B (eastern North America)	***genualis* Loew**
–	Intra-alar seta at transverse suture present (western North America)	**9**
9	Postcranial hairs (beard) with dorsal one-quarter or more brown to black; femur I with long (subequal to width of femur), white hairs ventrally to *pv*; wing as in Fig. 16A	***pterodactyl* sp. n.**
–	Postcranial hairs (beard) white or yellow, at most with a few black hairs dorsally; femur I with much shorter white hairs ventrally to *pv*	**10**
10	Wing with small brown cloud on or near M_1_ beyond crossvein dm-cu (this brown clouding sometimes faint) (Fig. 16B, D–G)	**11**
–	Wing without small brown cloud on or near M_1_ beyond crossvein dm-cu (Fig. 16C)	***hydrophilus* Aldrich**
11	Brown clouding in cell r_4+5_ more or less continuous with clouding in cell bm+dm (often joined by light brown clouding) (Fig. 16B, D, F)	**13**
–	Brown cloud in cell r_4+5_ separated from cloud in cell bm+dm by distinct clear space (Fig. 16E, G)	**12**
12	Wing with crossvein dm-cu meeting M_1_ at approximately 90° angle; brown cloud in cell bm+dm intersecting crossvein dm-cu (Fig. 16E)	***similis* Aldrich**
–	Wing with crossvein dm-cu meeting M_1_ at nearly 45° angle; brown cloud in cell bm+dm not reaching crossvein dm-cu (Fig. 16G)	***querulus* Osten Sacken**
13	Acrostichal setae stout, long (two-thirds to three-quarters size of dorsocentral setae); 3^rd^ costal sector (at apex of cell r_2+3_) about 1.5 times length of 4^th^ costal sector (at apex of cell r_4+5_) (Fig. 16D)	***limbatus* Van Duzee**
–	Acrostichal setae absent, or short and hair-like (less than one-half size of dorsocentral setae); 3^rd^ costal sector about 2 times length of 4^th^ costal sector (Fig. 16B, F)	**14**
14	Acrostichal setae short, with at least one seta posterior to anterior-most dorsocentral seta (Arizona, Mexico)	***sonorus* sp. n.**
–	Acrostichal setae absent, or minute and confined to anterior slope of scutum (eastern North America)	***genualis* Loew**

### Liancalus genualis

Taxon classificationAnimaliaDipteraDolichopodidae

Loew, 1861

**Figure 13.**
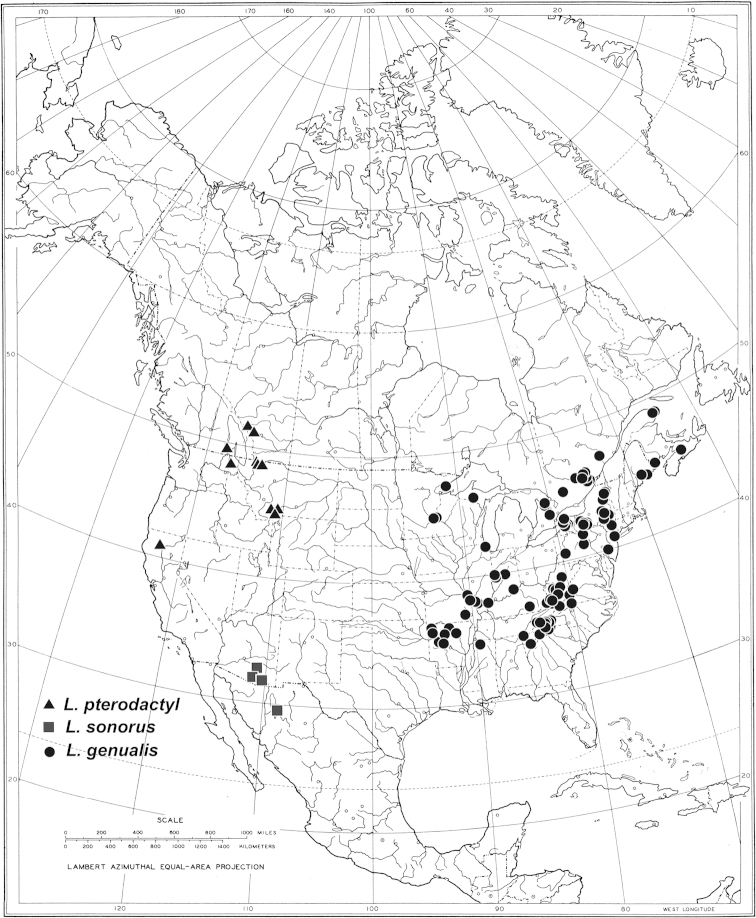
Known distributions of *Liancalus pterodactyl* sp. n., *Liancalus sonorus* sp. n., and *Liancalus genualis* Loew.

Figs 2A , 3C , 5A , 10A , 13 , 16B

#### Diagnosis.

Males and females are distinguished by the absence of acrostichal setae and having only 1 presutural intra-alar seta. Males are further distinguished by having tarsus I with tarsomere 2 very short (Fig. 3C), cerci long (Fig. 2A), and wing as in Fig. 5A. This species is most similar to *Liancalus sonorus* sp. n. but easily separated by the number of intra-alar seta (*Liancalus sonorus* have 2), the male wings (Fig. 5A–B), and distribution (Fig. 13).

#### Redescription.

**Male.** Body length 7.0–9.0 mm, wing length 6.5–7.5 mm. **Head:** Face narrowed below antenna, slightly widening toward palpus, green or blue with silver-gray pollen that is most dense along eyes and below frontoclypeal suture. Ommatidia near face slightly larger than remaining ommatidia. Vertex covered with silver pollen that is sparse medially revealing metallic green or blue ground color. Vertical setae on very small elevation; ocellar tubercle prominent with 2 large setae (slightly larger than vertical setae); with 2 postocellar setae which are two-thirds length ocellar setae; postocular setae approximately one-third size of vertical setae with about dorsal one-half black (approximately 12 black setae), ventral half (approximately 15 setae) white and more slender and slightly longer than black postocular setae. Ventral postcranial hairs (beard) rather sparse, wholly white. Palpus black, covered with moderately dense silver pollen and long, dense, dark brown to black setae on basal half. Antenna black, first flagellomere about as long as wide, broadly pointed apically, arista inserted near midpoint of dorsal edge.

**Thorax:** Scutum green to green-blue with moderately dense silver-gray pollen, with large red-bronze stripes between dorsocentral setae, and along intra-alar setae; medial stripe metallic pink; posterior slope of scutum bronze with lateral blue-green spots; scutellum bronze, with sparse silver pollen; notopleuron and postpronotum covered with silver pollen, often with some blue-green reflections; scutum with acrostichal setae absent (sometimes with a few small hairs on anterior slope of scutum); 6 dorsocentral setae, 2 notopleural setae, 1–2 strong, black postpronotal setae (often a few smaller white hairs), 1 presutural intra-alar seta (the posterior-most seta near transverse suture missing but with remnant dark spot where this seta inserted in other *Liancalus* – similar to small darkened area surrounding insertion of other setae on dorsum), 1 presutural seta, 2 postsutural supra-alar setae, and 1 postalar seta per side; scutellum with 6 large marginal setae (3 per side), no additional hairs; proepisternum with 1 dorsal and 1 ventral tuft of white hairs. Pleura metallic bronze-green, covered with dense silver-gray pollen, without setae or hairs.

**Legs:** Legs concolorous with pleura, but with distinctly less silver-gray pollen, femoral ‘knees’ orange (Fig. 2A). Coxa I uniformly covered with white hairs on anterior surface (length of hairs subequal to width of coxa I), with a few black, slender setae at apex. Coxa II with a few white hairs anteriorly, a few white setae near apex, and a black *ad* seta just beyond 1/2. Coxa III with a few short, white hairs on anterior surface and a black dorsal seta near 1/2. Femur I with sparse, short, white hairs *av* to *pv* on basal half (length < half width of femur). Femur II with row of short (≤ width of femur) posterior to *pv* setae on distal half, those near to just beyond middle of femur white, longest and becoming black and shorter apically. Femur III with some white hairs (length ≤ width of femur) on dorsal and posterior surface at base. Tarsus I(1) long, as long as remaining tarsomeres combined (Fig. 3C). Tarsus I(2) very short, about as long as wide, with ventral row of setae/setulae (Fig. 3C). Ratios of tibia:tarsomeres for leg I: 18-9-1-4-3-2; for leg II: 28-25-11-4-2-2; for leg III: 35-18-19-5-2-2.

**Wing** (Fig. 5A): Hyaline, with a longitudinal spurious vein between R_4+5_ and M_1_ that is arched on apical third of wing and terminates near midpoint of a circular, translucent, apical spot that is white in certain lights; this spot enclosed within a brown, apical cloud that extends anteriorly along costa to R_1_; some light clouding also evident narrowly along R_4+5_ on apical one-quarter of wing. Wing margin excavated posterior to M_1_; with short, broad lobe between M_1_ and CuA_1_. Calypter yellow with a fan of long, pale yellow setae at apex. Halter pale yellow.

**Abdomen:** Cylindrical, elongate, rather blunt at apex (Fig. 2A); T1 metallic green with dense silver pollen laterally becoming less dense dorsally, with bronze along posterior edge and occasionally with a diffuse bronze stripe dorsally. T2-T4 blue-green with dense silver pollen on basal one-half to two-thirds, with apical one-third to one-half bronze. T5 dark bronze with metallic green reflections and sparse silver pollen. T6 dark bronze with blue-green reflections and with dense silver pollen. T1-T3 with yellowish hair laterally, longest on T1 and T2, without black hairs or setae. Sternites bronze with dense silver-gray pollen. S1 bare except for lateral small tuft of yellow hairs at extreme base. S2 and S3 with sparse yellow hairs. S4 mostly bare. Hypopygium (Fig. 10A): cercus very long, slender, cylindrical, with long pale yellow dorsal and ventral setae (Fig. 2A).

**Female.** Body length 6.0–7.5 mm, wing length 6.0–7.0 mm. Similar to male except for face wider, dark violet-green covered with moderate to dense brown pollen; palpus black with silver pollen apically, dense golden-brown pollen basally, and black setae. Femur II posteriorly to *pv* with row of short (< half width of femur) hairs on apical half, those near middle white and becoming black apically. Wing (Fig. 16B) hyaline, with three diffuse brown clouds: largest one in cells r_4+5_ and bm+dm and crossing M_1_ near midpoint of wing, one on M_1_ beyond crossvein dm-cu, and smallest one on R_4+5_ at apex of wing.

#### Remarks.

Loew reported types from "Middle States"; a hand-written label on one of the paralectotypes collected by Loew appears to be "KY". Adults of this species have been frequently found in caves, e.g., in Arkansas (Barnes et al. 2009), Indiana (Banta 1907), Georgia (Reeves et al. 2000), Kentucky and Minnesota (see material examined).

#### Distribution.

This is the only species of *Liancalus* known to occur in eastern North America (Fig. 13).

#### Type material examined.

**LECTOTYPE** (designated here to fix identity of the species) ♂, labelled: "Loew/ Coll."; "Type 12944" [red label]; "Jan.-Jul. 2007/ MCZ Image/ Database" [with image of a camera]; "MCZ-ENT/ 00012944" [with barcode]; "LECTOTYPE/ ♂ Liancalus/ genualis Loew/ des. Runyon & Hurley" [red label] (MCZ). **PARALECTOTYPES:** Same data as lectotype, but without MCZ Image Database label, MCZ-ENT 00302768 (1 ♂, MCZ). Same data as previous, MCZ-ENT 00302769 (1 ♀, MCZ). Same data as previous, MCZ-ENT 00302770 (1 ♀, MCZ). Same data as previous, "KY" [?]; "Liancalus genualis" [hand-written]; MCZ-ENT 00302767 (1 ♂, MCZ).

#### Additional material examined.

**CANADA.** **New Brunswick:** St. Andrews, littoral rocks, 19.v.1978, S.A. Marshall (1 ♀, CNC). **Nova Scotia:** Truro, 5.vii.1913, R. Matheson (1 ♀, CUIC). **Ontario:** Ottawa, 1.viii.1926, C.H. Curran (1 ♀, CNC), Ottawa, 12.viii.1924, F.P. Ide (1 ♀, CNC), Ottawa, 22-30.ix.1956, JRV (5 ♂, 19 ♀, CNC), same as previous, at seepage on limestone cliff, 1-7.viii.1987, JRV (18 ♂, 7 ♀, CNC), same as previous, 2.viii.1988 (6 ♂, 6 ♀, CNC), same as previous, 15-21.vii.1956 (7 ♀, CNC), same as previous, 15-16.viii.1956 (1 ♂, 2 ♀, CNC), Niagara Falls, 6.viii.1929, ALM (1 ♀, USNM), Owen Sound, 18.viii.1976, J.M. Cumming (1 ♂, DEBU), Elora, 15.v.1977, K. Barber (2 ♀, DEBU, Inglis Falls, seepage spring, 11.vii.1985, B. Sinclair (1 ♀, DEBU), same as previous, madicolous zone, 25.vii.1985 (1 ♀, DEBU), Algonquin Park, 10.viii.1940, J.S. Rogers (1 ♂, UMMZ). **Quebec:** Gatineau Pk., Luskville Falls, 14.vii.1972, F. Brodo (2 ♂, CNC), Duncan L, nr. Rupert, 1.viii.1969, J.F. McAlpine (1 ♂, 1 ♀, CNC), Gaspe Bay, 18.vii.1931, JMA (2 ♀, USNM), Gatineau Pk., Lusk Falls, 26.vi.1991, JRV (1 ♂, 1 ♀, CNC), Wakefield, 1.viii.1959, JRV (1 ♀, CNC), Lac Mondor, Ste. Flore, 6.v.1951, E.G. Munroe (1 ♀, CNC), Meach L, Old Chelsea, 16.iv.1973, D.M. Wood (2 ♀, CNC), Old Chelsea, 20.ix.1955, JRV (1 ♀, CNC), same as previous, 9.x.1955 (1 ♀, CNC), same as previous, 16.v.1958 (1 ♀, CNC), Gaspe, 24.viii.1937, C.P. Alexander (1 ♂, EMUS), Kingsmere, 16.v.1958, J.G. Chillcott (1 ♀, CNC), Vinton, 5-12.vi.1900 (2 ♂, OSU), Kazubazua, 16.viii.1927, F.P. Ide (1 ♀, CNC). **USA.** **Arkansas:** Springdale, viii.1933 (1 ♀, MCZ); Logan Co., Magazine Mt, Ozark For., 2800', 11.vi.1948, S.S. Roback (10 ♂, 11 ♀, INHS); Marion Co., Buffalo R, 670-800', 30.vii.1974, G.W. Byers & C.W. Young (7 ♂, 6 ♀, SEMC); Pope Co., Ozark Nat. For., Falling Water Falls, 22.v.1991, J.E. Swann (1 ♀, DEBU); Stone Co., Ozark Nat. For., Hwy 5, roadcut seepage, 21.v.1991, B.J. Sinclair (2 ♂, 2 ♀, CNC); Washington Co., Devil’s Den St. Park, 1400 ft, 27.v.1977, Byers, May & Young (1 ♂, 1 ♀, SEMC); Yell Co., Mt. Nebo St. Pk., ex. Gum Sprg. Tr., 22.v.1991, B.J. Sinclair (1 ♂, CNC). **Kentucky:** Whitley Co., Cumberland Falls St. Pk., ex. roadcut seeps, 17-18.vi.1990, B.J. Sinclair (1 ♂, CNC). **Georgia:** Neels Gap, 5.vi.1946, P.W. Fattig (1 ♂, 1 ♀, USNM); Bartow Co., Yarborough Cave (Adairsville), ix.1998, W. Reeves (1 ♂, MTEC); Dade Co., Cloudland Canyon S.P., ex. limestone seep, 15.v.1986, B.J. Sinclair (1 ♂, 1 ♀, DEBU). **Illinois:** Giant City, 28.vii.1930, Knight & Ross (4 ♂, 4 ♀, INHS); Jackson Co., Makanda, 26.vi.1909, sweeping (5 ♂, 2 ♀, EMUS, INHS), same as previous, 4.vi.1919 (2 ♀, INHS); Lake Co., Lake Forest, 12.v.1904, J.G.N. (1 ♂, 6 ♀, CUIC); Pope Co., Herod, 9.viii.1905 (7 ♂, 5 ♀, INHS). **Indiana:** Jefferson Co., Hanover, Crowe Ravine, 11.vi.1921, C.P. Alexander (1 ♂, USNM), Clifty Falls St. Pk., interior of old tunnel, 5.ix.1950, T.H. Hubbell (1 ♂, UMMZ); Marion Co., vic. Indianapolis, 1.viii.1943, FCH (9 ♂, 9 ♀, CMNH, CNC, EMUS, FSCA, MTEC), same as previous, 8.viii.1943 (2 ♂, 2 ♀, CAS, FSCA), same as previous, 22.viii.1943 (2 ♂, 2 ♀, CAS, MTEC), same as previous, 4.vi.1944 (8 ♂, 5 ♀,CAS, CNC, EMUS, FSCA, LACM, OSAC), same as previous, 11.vi.1944 (2 ♂, 2 ♀, CNC, INHS); Vigo Co., Terre Haute, 6.viii.1944, FCH (15 ♂, 15 ♀, CUIC, EMUS, FSCA, INHS, ISUI, MTEC, NYSM), Nr. Ft. Harrison, 11.vii.1943, FCH (4 ♂, 5 ♀, CMNH, EMUS, FSCA, MTEC). **Maine:** Acadia Nat. Pk., 3.viii.1972, FCH (2 ♂, 6 ♀, CAS, EMUS, FSCA); Waldo Co., Belfast, 2.viii.1972, FCH (1 ♂, MTEC). **Michigan:** Iron Co., T43N-R35W-Sec. 24, 27.vii.1971, DDW (4 ♂, 6 ♀, MSUC, USNM). **Minnesota:** Hennepin Co., Minneapolis, O.W. Oestlund (1 ♀, UMSP), Fort Snelling, in sandstone cave, 9.i.1935, D.G. Denning (2 ♀, UMSP); Lake Co., Encampment R, 6.viii.1939, R.H. Daggy (3 ♀, UMSP), 5.viii.1939, H.T. Peters (1 ♂, UMSP); Ramsey Co., 20.v.1936, D. Denning (1 ♀, UMSP). **Mississippi:** Lafayette Co., Spring 1943, F.M. Hull (2♂, 6 ♀, CNC). **Missouri:** Saint Louis, 6.vii.1972, FCH (4 ♂, 1 ♀, CAS, EMUS, FSCA); Carter Co., Big Spring Park, 16.vii.1953, G.W. Byers (3 ♂, UMMZ); Sainte Genevieve Co., vi.1951, W. Downes (2 ♀, MTEC). **New Jersey:** Bergen Co., Palisades, 4.vii.1920, J. Bequaert (3 ♂, 1 ♀, MCZ, WSU). **New York:** J. Brown’s Well, Fulton Ck, 13.viii.1905, N.Y.S. Coll. (1 ♂, 4 ♀, CUIC); Albany Co., Meadowdale, 2.viii.1904, N.Y.S. Coll., D.B. Young (2 ♂, 9 ♀, CNC, NYSM); Dutchess Co., Po’k’psie, N.Y.S. Coll., 17.vi.1904 (2 ♂, 6 ♀, NYSM); Erie Co., Spring Brook, 25.vi.1911, M.C. Van Duzee (3 ♀, CAS), S. Wales, 9.vii.1911, M.C. Van Duzee (1 ♀, CAS), Colden, 3.viii.1913, M.C. Van Duzee (1 ♂, 1 ♀, CAS, NYSM), same as previous, 2.viii.1914 (1 ♀, CAS), same as previous, 9.viii.1914 (1 ♀, CAS), same as previous, 9.vii.1922 (2 ♂, 2 ♀, CAS); Essex Co., Keene Valley, around and on wet cliff face, 1200', 20.vii.1962, JRV (1 ♂, 8 ♀, CNC), Keen Valley (Beede’s), 3.viii.1886, N.Y.S. Coll. (1 ♂, 8 ♀, NYSM, USNM), same as previous, 5.viii.1886 (1 ♀, NYSM), same as previous, 7.viii.1889 (1 ♂, 6 ♀, NYSM, USNM), same as previous, 12.viii.1889 (2 ♂, NYSM), same as previous, 16.vii.1890 (1 ♀, NYSM), same as previous, 12.viii.1890 (1 ♂, NYSM, USNM), Elizabethtown, 22.viii.1937 (6 ♂, 5 ♀, MTEC); Fulton Co., Gloversville, 19.vi.1910, Alexander (1 ♀, CUIC); Greene Co., Prattsville, 3.viii.1974, FCH (1 ♀, INHS); Hamilton Co., 6 mi. E Indian L, 43°45'30", 74°10'14", 15.v.1977, 1820', T.L. McCabe (1 ♀, NYSM); Montgomery Co., Canajoharie, 8.vii.1934, H.K. Townes (2 ♀, AMNH); Niagara Co., Niagara Falls, 31.vii.1910, M.C. Van Duzee (6 ♂, 9 ♀, CAS, CNC, CUIC, OSU), same as previous, 4.viii.1912 (1 ♂, 1 ♀, CAS, OSU), same as previous, 6.x.1912 (1 ♂, CAS), same as previous, 19.v.1918 (2 ♀, CAS, NYSM), same as previous, 17.vii.1921 (2 ♂, 1 ♀, CAS); Tompkins Co., Ithaca, 5.viii.1947 (1 ♂, INHS), Ithaca, Buttermilk, 18.vii.1920 (2 ♂, 6 ♀, CUIC), Ludlowville, 4.vii.1965, L.L. Pechuman (1 ♀, CUIC), same as previous, 7.vii.1966 (1 ♀, CUIC), same as previous, 4.viii.1966 (1 ♂, CUIC), same as previous, 6.vii.1967 (1 ♀, CUIC), same as previous, 29.viii.1970 (1 ♀, CUIC), same as previous, 26-27.vi.1973 (2 ♀, CUIC), same as previous, 6-13.vii.1963 (3 ♀, CUIC, USNM), same as previous, waterfall, 13.vi.1979 (3 ♀, CUIC), Ithaca, 25.iii.1917, R.C. Shannon (2 ♀, USNM), Ithaca, 15.viii.1928, ALM (3 ♂, 2 ♀, USNM), Ithaca, 26.iii.1991 (2 ♀, USNM), Ithaca, 2.viii.1887, JMA (1 ♂, USNM), Ithaca, 26.iii.1891, JMA (3 ♀, USNM), Ithaca, 13.vii.1893, JMA (1 ♂, USNM), Ithaca, 24.vii.1894, JMA (1 ♂, USNM), Ithaca, vii.1901, JMA (2 ♂, 1 ♀, USNM), Ithaca, Sixmile Crk, 29.vii.1958, H.E. Evans (2 ♀, CUIC), same as previous, 9.viii.1961, J.L. Laffoon (2 ♂, 5 ♀, ISUI), Ithaca, Cornell U. campus, 2.iii.1972, G. & K. Eickwort (1 ♀, CUIC), Taughanic, Ithaca, 21.iv.1917, Shannon (3 ♀, CUIC), Taughannock Falls, 21.iv.1917, S.H. Emerson (1 ♀, CUIC), same as previous, E.G. Anderson (2 ♀, UMSP), Ithaca, 31.iii.1917, S.H. Emerson (1 ♀, UMSP), Ithaca, Coy Glen, 9.viii.1961, J.L. Laffoon (1 ♂, 1 ♀, ISUI), Ithaca, 4.iv.1924, C.H. Curran (1 ♀, AMNH), Ithaca, 22-25.iii.1917, E.G. Anderson (9 ♀, UMSP), same as previous, 18.iv.1917 (3 ♀, CUIC, UMSP), Ithaca, 25.iii.1915, R.C. Shannon (2 ♀, CUIC), same as previous, 25.iii.1917 (9 ♀, CUIC); Tompkins Co., Ludlowville, 4.vii.1965, L.L. Pechuman (1 ♀, CUIC), same as previous, 7.vii.1966 (1 ♀, CUIC), same as previous, 4.viii.1966 (1 ♂, CUIC), same as previous, 6.vii.1967 (1 ♀, CUIC), same as previous, 29.viii.1970 (1 ♀, CUIC), same as previous, 26-27.vi.1973 (2 ♀, CUIC), same as previous, 6–13.vii.1963 (3 ♀, CUIC, USNM), same as previous, waterfall, 13.vi.1979 (3 ♀, CUIC), Ithaca, Buttermilk, 18.vii.1920 (2 ♂, 6 ♀, CUIC), Ithaca, Coy Glen, 9.viii.1961, J.L. Laffoon (1 ♂, 1 ♀, ISUI), Ithaca, Sixmile Crk, 29.vii.1958, H.E. Evans (2 ♀, CUIC), same as previous, 9.viii.1961, J.L. Laffoon (2 ♂, 5 ♀, ISUI), Ithaca, Cornell U. campus, 2.iii.1972, G. & K. Eickwort (1 ♀, CUIC), Taughanic, Ithaca, 21.iv.1917, Shannon (3 ♀, CUIC), Taughannock Falls, 21.iv.1917, S.H. Emerson (1 ♀, CUIC), same as previous, E.G. Anderson (2 ♀, UMSP), Ithaca, 2.viii.1887, JMA (1 ♂, USNM), same as previous, 26.iii.1891 (3 ♀, USNM), same as previous, 13.vii.1893 (1 ♂, USNM), same as previous, 24.vii.1894 (1 ♂, USNM), same as previous, vii.1901 (2 ♂, 1 ♀, USNM), same as previous, 25.iii.1915, R.C. Shannon (2 ♀, CUIC), same as previous, 25.iii.1917 (11 ♀, CUIC), same as previous, 22-25.iii.1917, E.G. Anderson (9 ♀, UMSP), same as previous, 18.iv.1917 (3 ♀, CUIC, UMSP), same as previous, 31.iii.1917, S.H. Emerson (1 ♀, UMSP), same as previous, 4.iv.1924, C.H. Curran (1 ♀, AMNH), same as previous, 15.viii.1928, ALM (3 ♂, 2 ♀, USNM), same as previous, 5.viii.1947 (1 ♂, INHS), same as previous, 26.iii.1991 (2 ♀, USNM); Yates Co., Penn Yan, 30.vii.1972, FCH (1 ♂, 1 ♀, CAS). **North Carolina:** Mt. Pisgah, 4-5000 ft, 8.vii.1959, H.V. Weems Jr. (3♂, 5 ♀, FSCA); Haywood Co., Wagon Tree Gap, Blue Ridge Pkwy, 30.v.1965, J.G. Chillcott (1 ♂, 1 ♀, CNC), 19 mi. S Canton, 2.viii.1988, RLH (2 ♀, MTEC); Macon Co., Highlands, 17.vi.1957, JRV (1 ♀, CNC); Swain Co., GtSmokies NP, Clingmans Dome, 21.vi.1941, ALM (1 ♂, 2 ♀, USNM), Nantahala Gorge, 2000 ft, 27.viii.1930, N. Banks (1 ♀, MCZ); Transylvania Co., Near L Toxaway, 3000 ft, 28.viii.1930, Carpenter (1 ♀, MCZ), "Tennessee Ridge" Mts, Owen’s Gap, 4000 ft, 28.viii.1930, N. Banks (1 ♀, MCZ); Yancey Co., Black Mts, Mt Mitchell, 5000–6711 ft, ix.1930, N. Banks (1 ♂, MCZ). **Pennsylvania:** Allegheny Co., Carn. Mus., on window, 5.x.1903, H. Kahl (1 ♂, CMNH); Lycoming Co., 3 mi. S Ralston, 4.vi.1983, RLH (1 ♀, MTEC), 27.vii.1972, DDW (2 ♀, CAS); Mifflin Co., 0.4 mi. W Laurel Crk Rsvr, 1240 ft, 28.viii.2003, JBR (1 ♂, 1 ♀, MTEC); Philadelphia Co., West Park, Phila, 21.viii.1894, JMA (2 ♀, USNM), same as previous, C.W. Johnson (1 ♂, EMUS), same as previous, Philadelphia (3 ♀, MCZ). **South Carolina:** Pickens Co., Table Rock State Park, 35°2'N, 82°32'W, 1500', 6.vii.1958, J. Laffoon (6 ♂, 8 ♀, ISUI). **Tennessee:** Smoky Mts, 5800 ft, 6.vi.1939, C.P. Alexander (1 ♂, EMUS). **Virginia:** Botetourt Co., North Crk Area, 21.ix.1983, Kondratieff (1 ♂, 2 ♀, CSUC), near Arcadia, 21.ix.1983, S. Bullington (3 ♂, CSCA); Dickenson Co., Ramey Fork, 3 mi. N Clintwood, 1400 ft, 15.viii.2008, JBR (3 ♂, 1 ♀, MTEC), Pound R, N37°11.58', W82°26.63', 4.vi.2008, JBR (3 ♂, 2 ♀, MTEC), Mill Crk, 0.5 mi. NW Blowing Rock, 1900 ft, N37°1.929', W82°25.514', 16.vi.2008, JBR (1 ♂, MTEC); Grayson Co., Mt Rogers S slope, 13.vii.1969, J.B. Karren (1 ♀, EMUS); Montgomery Co., Cedar Run – RR tracks, 13.vii.1979, P. Firth (1 ♀, CSUC); Patrick Co., Pinnacles of Dan, 15.iv.1978, B. Kondratieff (1 ♀, CSUC). **West Virginia:** Boone Co., Hwy 3 at Lincoln Co. line, 3.viii.1992, RLH (1 ♂, MTEC); Braxton Co., 1.5 km NW Falls Mill, Little Kanawha R, seep, 14.xi.2003, D.R. Jones & N.L. Smith (4 ♀, MTEC); Logan Co., 2 mi. SE Blair, 3.viii.1992, RLH (2 ♂, 1 ♀, MTEC); Nicholas Co., Gauley R, Summerville Dam, 1400 ft, 11.v.2004, JBR (1 ♂, 3 ♀, MTEC); Wyoming Co., under bridge over creek, 14.vii.1982, Kondratieff (1 ♂, CSUC).

### Liancalus hydrophilus

Taxon classificationAnimaliaDipteraDolichopodidae

Aldrich, 1893

**Figure 14.**
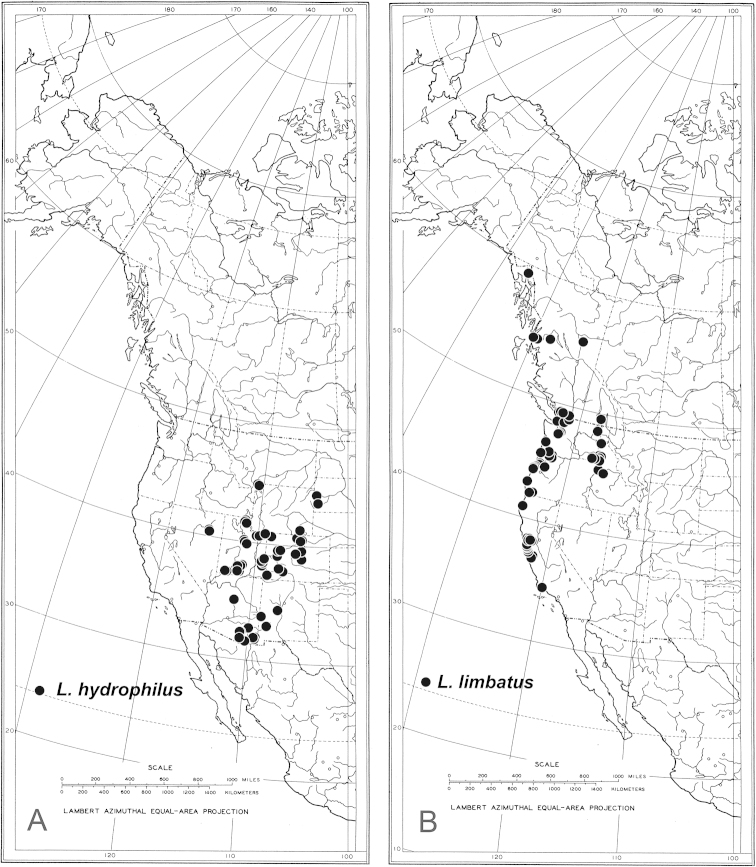
Known distributions of **A** *Liancalus hydrophilus* Aldrich and **B** *Liancalus limbatus* Van Duzee.

Figs 3B , 4B , 8 , 11B , 14A , 16C

#### Diagnosis.

This is one of two Nearctic species in which males have setae on the wing margin (Fig. 4). Males can be distinguished by the relatively short, rounded lobe between wing veins M_1_ and CuA_1_ (Fig. 4B), and by the long cerci with evenly spaced, long setae along their full-length (Fig. 8).

#### Redescription.

**Male.** Body length 8.0–10.5 mm, wing length 6.5–8.0 mm. Habitus (Fig. 8). **Head:** Face rather broad, widening toward palpus, metallic green above frontoclypeal suture, covered with dense silver pollen below suture. Vertex covered with silver pollen that is often sparser medially revealing metallic green-blue ground color. Vertical setae on very small elevation; ocellar tubercle prominent with 2 large setae (slightly larger than vertical setae); with 2 postocellar setae which are two-thirds length ocellar setae; postocular setae approximately one-half size of vertical setae with about dorsal one-half of postocular setae black (approximately 15 black setae), ventral half (approximately 10 setae) white and more slender and slightly longer than black postocular setae. Ventral postcranial hairs (beard) rather sparse, usually wholly white (sometimes dorsal-most 1–3 hairs brown to black). Palpus black, covered with moderately dense silver pollen apically, golden brown pollen at base, with long, black setae on basal two-thirds. Antenna black, first flagellomere about as long as wide, slightly pointed apically, arista inserted near midpoint of dorsal edge.

**Thorax:** Scutum green to green-blue with moderately dense silver-gray pollen, with red-bronze stripes between acrostichal and dorsocentral setae, and along intra-alar setae; posterior slope of scutum blue-green with medial bronze stripe; notopleuron and postpronotum (humerus) covered with dense silver pollen, often with some blue-green reflections; 6 dorsocentral setae; 3–8 acrostichal setae (usually < 6), in a single row; 2 notopleural setae; postpronotum with 1–3 strong, black setae and often a few smaller white hairs; 2 presutural intra-alar seta (one near suture); 1 presutural and 2 postsutural supra-alar setae; 1 postalar seta; scutellum mostly bronze, with some green-blue color at base, covered with sparse silver pollen, with 6 large marginal setae (3 per side), no additional hairs; proepisternum with 1 dorsal and 1 ventral tuft of white hairs. Pleura metallic bronze-green, covered with dense silver-gray pollen, without setae or hairs.

**Legs:** Legs concolorous with pleura, but with slightly less silver-gray pollen, femoral ‘knees’ very narrowly orange. Coxa I uniformly covered with white hairs on anterior surface (length of hairs subequal to width of coxa I), with a couple to few black, slender setae at apex. Coxa II with scattered white hairs anteriorly, a few white and black setae near apex, and a black *ad* seta just beyond 1/2. Coxa III with a few short, white hairs on anterior surface and a black dorsal seta near 1/2. Femur I with row of black, *pd* setae on apical half (length ≤ half width of femur). Femur II with row of posterior setae on distal half (length ≤ width of femur), those near to just beyond middle of femur longest, white, and becoming black and shorter apically. Femur III with scattered white hairs (length ≤ width of femur) dorsally at base. Tarsus I(2) very short, about as long as wide, with ventral row of setae/setulae (Fig. 3B). Ratios of tibia:tarsomeres for leg I: 17-7-2-5-3-2; for leg II: 24-20-8-4-2-2; for leg III: 35-17-13-4-2-2.

**Wing** (Fig. 4B): Hyaline, with a longitudinal spurious vein immediately above M_1_ that ends near junction of M_1_ with dm-cu and usually two small brown clouds just beyond junction of dm-cu and CuA_1_ (these spots sometimes faint); apical portion of membrane between R_4+5_ and M_1_ with a small, narrow, translucent, area that is white in certain lights and enclosed within a small, brown cloud. Fourth costal sector (between R_4+5_ and M_1_) flattened with a cluster of 3–6 black setae at apex of M_1_ that are usually fused apically; wing margin between M_1_ and CuA_1_ with a rather short (length subequal to width), broad lobe bearing several black setae at apex that are usually fused apically. Calypter yellow with a fan of long, pale yellow setae at apex. Halter pale yellow.

**Abdomen:** Cylindrical, elongate, rather blunt at apex (Fig. 8); T1 metallic green with dense silver pollen laterally becoming less dense dorsally, with bronze along posterior edge and occasionally with a diffuse bronze stripe dorsally. T2-T4 green-blue with dense silver pollen on basal one-half to two-thirds, with apical one-third to one-half bronze; T2 usually bronze dorsally at base. T5 dark bronze with metallic green reflections and sparse silver pollen. T6 dark bronze with blue-green reflections and dense silver pollen. T1-T3 with wholly to nearly wholly yellowish hair laterally, longest on T1 and T2. Sternites bronze with dense silver-gray pollen. S1 bare except for lateral small tuft of yellow hairs at extreme base. S2 and S3 with sparse yellow hairs. S4 mostly bare. Hypopygium (Fig. 11B): cercus very long, as long or longer than abdomen, slender, cylindrical, with very long, evenly-spaced, pale yellow setae along full-length ventrally (Fig. 8), with some hairs crinkly on apical half.

**Female.** Body length 6.0–8.0 mm, wing length 6.0–7.0 mm. Similar to male except for face wider, dark metallic green to violet covered with sparse to moderately dense brown pollen; palpus black with silver pollen along margins and dense brown pollen basally, and black setae. Femur I with *pd* row of black setae on apical half (longest subequal to width of femur) and very sparse, very short white hairs on posterior and ventral surface. Femur II posteriorly to *pd* with row of short (length ≤ width of femur) hairs which are white on basal half and black on apical half. Wing (Fig. 16C) hyaline, with a rather large, diffuse, faint brown cloud in cells r_4+5_ and bm+dm and crossing M_1_ just beyond midpoint of wing; usually with some indistinct brown clouding along wing margin between R_1_ and M_1_.

#### Remarks.

Aldrich (1893) described the habits of this species at the type locality near Custer, SD. On page 570, Aldrich writes, "From several seams in the rock there is a gentle flow of almost ice-cold water, which covers a considerable area of vertical rock in a thin sheet. Standing in this icy water were my flies!" Similarly, Van Duzee (1917: 127) wrote, "I took a single male…resting on rocks over which water was trickling in South Cheyenne Canon [sic]".

#### Distribution.

This species is confined to interior western North America (Fig. 14A).

#### Type material examined.

**LECTOTYPE** (designated here to fix identity of the species) ♂, labelled: "Custer/ SD"; "JM Aldrich/ Coll"; "COTYPE/ No. 50276/ U.S.N.M." [red label]; "Liancalus/ hydrophilus/ Ald." [hand-written]; "LECTOTYPE/ ♂ Liancalus/ hydrophilus Aldrich/ des. Runyon & Hurley" [red label] (USNM). **PARALECTOTYPE:** ♂, labelled: "Cotype" [hand-written]; "Custer SD"; "det. Aldrich"; "Type 13503" [red label]; "PARALECTOTYPE/ ♂ Liancalus/ hydrophilus Aldrich/ des. Runyon & Hurley" [red label] (MCZ).

#### Additional material examined.

**USA.** **Arizona:** Cochise Co., 18-28.vi.1916, V.W. Owen (4 ♂, 6 ♀, CAS), Portal, S.W. Res. Sta. AMNH, 23.vi.1968, V.D. Roth & FCH (1 ♂, MTEC), Rustler Park, 9.vi.1972, W.W. Wirth (1 ♂, 1 ♀, USNM), same as previous, 5.vii.1940, R.H. Beamer (1 ♀, SEMC), Ramsey Cyn, Huachuca Mts, 5500 ft, 23.iv.2002, RLH & JBR (3 ♀, MTEC), Cave Crk at Herb Martyr Campgrd (Dam), 5800 ft, N31°52.35', W109°14.09', 6.iv.2011, JBR (1 ♂, 1 ♀, MTEC), Chiricahua Mts, Rustlers Pk., 5.vii.1940, D.E. Hardy (1 ♀, SEMC); Coconino Co., Oak Crk Cyn, 24 mi. S Flagstaff, 25.vii.1959, Samuelson & Radford (1 ♀, UAIC), Oak Crk Cyn, 3500', G.C. Eickwort (2 ♀, MSUC); Graham Co., Wet Cyn, Pinaleno Mts, 5900 ft, 26.iv.2001, RLH & JBR (1 ♂, 1 ♀, MTEC); Pima Co., Catalina Mts, Marshall Gulch near Summerhaven, 28.v.1986, J. Jenkins (15 ♂, 15 ♀, MTEC), Mt Lemmon, Catalina Mts, 14.vi.1979, M. Hetz (1 ♂, UAIC), Catalina Mts, HkHy mi22, 9.vi.1958, Nutting & Butler (1 ♀, UAIC); Santa Cruz Co., Coronado National Forest, Santa Rita Mts, Madera Cyn, 3–4.vi.1991, B.J. Sinclair (2 ♂, 1 ♀, CNC), same as previous, 27.iv.1979, K.N. Barber (1 ♂, 1 ♀, DEBU), same as previous, 5700 ft, N31°42.59', W110°52.18', 4.iv.2011, JBR (1 ♀, MTEC). **Colorado:** Green Mt Falls, 12.viii.1943, H.H. Ross (1 ♀, INHS); Boulder Co., Boulder (1 ♂, 1 ♀, CUIC), same as previous, JMA (2 ♂, 2 ♀, USNM), same as previous, C.W. Johnson (3 ♂, 1 ♀, MCZ); Cameron Pass, 13.viii.1964, FCH (2 ♂, 2 ♀, FSCA), Cameron Pass, Campbell L, 12.viii.1947, H.H. Ross (2 ♂, INHS); Garfield Co., Rifle, 18.vii.1947, FCH (2 ♂, 3 ♀, CAS, MTEC, ODAC), Glenwood Cyn, off I-70 rest stop, 19.ix.1986, M.K. Kroening (1 ♂, 1 ♀, CSUC); Larimer Co., Lory St. Park, 15.iv.1984, P. Opler (2 ♀, CSUC), Buckhorn Crk, 12.ix.1996, B. Kondratieff (1 ♂, CSUC); Mesa Co., Big Crk at jct. with Plateau, Rt. 330, 19.ix.1986, M.K. Kroening (1 ♂, CSUC); Mineral Co., Hwy 160, Treasure Falls, 15.viii.1983, PHA (4 ♀, CAS); Moffat Co., 11.vii.1986, Yampa R, B. Kondratieff (1 ♂, CSUC); Montezuma Co., Dolores, 9.vi.1960, FCH (1 ♂, 1 ♀ FSCA); Ouray Co., 5 km S of Ouray, on Uncompahgre R, 2697 m, 14.viii.1973, PHA (1 ♂, CAS); Park Co., Fairplay, 13.vi.1961, FCH (1 ♂, 2 ♀, FSCA, MTEC). **Nevada:** Elko Co., Lamoille Crk, 25 mi. SE Elko, 8800 ft, 11.viii.2005, JBR & RLH (1 ♂, 3 ♀, MTEC), Ruby Mts, W of Terrace Cmpgrd, 16.viii.1989, RLH (1 ♂, 1 ♀, MTEC). **New Mexico:** Catron Co., Luna, 25.vi.1968, FCH (4 ♂, 5 ♀, CAS, EMUS, FSCA), Reserve, 25.vi.1968, FCH (1 ♂, 1 ♀, CAS, FSCA); Grant Co., Silver City, 25.vi.1968, FCH (1 ♀, MTEC); Socorro Co., Magdalena Mts, viii.1894, H. Kahl (2 ♂, 1 ♀, CMNH), Magdalena, W.M. Wheeler (2 ♂, 2 ♀, AMNH, CAS). **South Dakota:** Custer Co., Custer (5 ♂, 8 ♀, LACM, UMSP, USNM), French Crk, Black Hills, A. Borkent, 25.iv.1981 (2 ♀, CNC); Lawrence Co., Bridal Veil Falls, 6 mi. S Spearfish, 4250 ft, 19.viii.2003, JBR (2 ♂, 2 ♀, MTEC). **Utah:** Boulder Mt, 26.viii.1978, FCH (14 ♂, 7 ♀, CAS, EMUS, FSCA, MTEC), Zion Pk, 29.vi.1940, ALM (1 ♂, USNM); Cache Co., Logan Meadows, 15.x.1939, G.F. Knowlton & FCH (2 ♂, 2 ♀, CAS, CNC), Logan Cyn, 28.viii.1939, FCH (3 ♂, 2 ♀, CAS, EMUS, OSU), same as previous, 15-18.viii.1940 (2 ♂, 5 ♀, EMUS), same as previous, 24.x.1969, R. Kirkland (1 ♂, BYU), Willow Park, 23.viii.1986, B.C. Giwod (1 ♂, EMUS), Logan, 3-11.1934, C.F. Smith (1 ♀, AMNH), same as previous, T.O. Thatcher (1 ♀, AMNH); Daggett Co., Linwood, 6.ix.1939, G.F. Knowlton & FCH (1 ♂, 1 ♀, CNC), Dowd Springs, 17.viii.1978, FCH (1 ♂, 8 ♀, MTEC); Duchesne Co., Uinta Canyon, Wandin Park 18.vii.1950, FCH (1 ♀, CNC), Pole Crk, Uinta Mts, 19.vii.1950, FCH (1 ♂, FSCA); Garfield Co., Death Hollow jct., Boulder Mail Trail, 37°50'37N, 111°30'52W, 11.vii.2000, E.C. Green, W.N. Mendel, M. Moody, C.R. Nelson (1 ♀, BYU), Grand Staircase-Escalante Natl. Monument, spring off Hwy 12, 7.5 mi. E Henrieville, N 37°36'46", W 111°53'48", 6578 ft, malaise trap, 2-17.viii.2000, W.N. Mendel, E.C. Green, M. Moody (2 ♂, BYU, MTEC), Calf Crk between upper and lower falls, off Hwy 12, 37°51'18N, 111°27'07W, E.C. Green, W.N. Mendel, C.R. Nelson (2 ♂, 1 ♀, BYU); Grand Co., Moab, 4.vi.1940, G.F. Knowlton & FCH (3 ♂, 3 ♀, FSCA, MTEC), same as previous, 18.v.1939 (1♂, 4 ♀, CAS, EMUS), La Sal Mts, Warner Cp., 9500', 21.vii.1968, Malaise trap, W.J. Hanson (1 ♀, EMUS), Westwater, 26.ix.1939, G.F. Knowlton & FCH (1 ♀, INHS); Kane Co., Sheep Crk jct., Skutumpah Rd, 37°29'43N, 112°03'59W, 5531 ft, 29.ix.1999, R.W. Baumann, K.T. Huntzinger, C.R. Nelson (1 ♂, MTEC), Snake Crk above jct. Paria R, 37°20'44N, 112°00'29W, 3-4.viii.2000, E.C. Green, W.N. Mendel, M. Moody, C.R. Nelson (1 ♂, BYU); San Juan Co., La Sal, 27.vi.1951, FCH (3 ♂, 6 ♀, CAS, EMUS, FSCA, CUIC); Utah Co., Mt Timpanogos, 4.viii.1946, FCH (3 ♂, 1 ♀, CNC, LACM), same as previous, 12.vii.1941, FCH & G.F. Knowlton (1 ♂, 1 ♀, EMUS), American Fork, 6.vii.1939, G.F. Knowlton (1 ♂, EMUS), St. George, 20.iv.1939, G.F. Knowlton & FCH (1 ♀, EMUS), Battle Crk, nr. Pleasant Grove, 31.vii.1984, C.R. Nelson (1 ♀, BYU), 12 mi. E Thistle, Hwy 6, 14.viii.1984, Nelson & Allred (2 ♂, 1 ♀, BYU); Washington Co., Zion Nat’l Park, Birch Crk, 27.vii.1975, W.J. Hanson (2 ♂, 5 ♀, EMUS), Zion Nat’l Park, narrows, 21.vi.1981, C.R. Nelson (1 ♂, 2 ♀, BYU). **Wyoming:** Albany Co., Laramie, 15.viii.1950, N. Davis (1 ♀, ISUI); Park Co., Yellowstone Nat’l Park, Rustic Falls, 7200 ft, 44°56.03' N, 110°43.55'W, 28.viii.2009, JBR (1 ♀, MTEC), Yellowstone Park, falls, 23.vii.1923, ALM (2 ♀, USNM).

### Liancalus limbatus

Taxon classificationAnimaliaDipteraDolichopodidae

Van Duzee, 1917

Figs 2C , 3D , 5C , 10C , 14B , 16D

#### Diagnosis.

Males of this species have long cerci, wing without marginal setae but with a distinct marginal lobe between veins M_1_ and CuA_1_, and with crossvein dm-cu relatively straight (Fig. 5C). Female wing as in Fig. 16D.

#### Redescription.

**Male.** Body length 8.0–9.0 mm, wing length 6.5–7.5 mm. **Head:** Face narrow, slightly widening toward palpus, metallic green to bronze with sparse to moderately dense silver pollen. Vertex covered with silver pollen mostly hiding metallic green to blue ground color. Vertical setae on very small elevation; ocellar tubercle prominent with 2 large setae (subequal in size to vertical setae); with 2 postocellar setae which are one-half to two-thirds size of ocellar setae; postocular setae approximately one-half size of vertical setae with about dorsal one-half of postocular setae black (approximately 15 black setae), ventral half (approximately 10 setae) white and more slender and slightly longer than black postocular setae. Ventral postcranial hairs (beard) rather sparse, usually wholly white but rarely with 1–3 brown to black setae. Palpus relatively small, black, covered with sparse silver pollen and scattered yellow to yellow-brown hairs. Antenna black, first flagellomere about as long as wide, rounded apically, arista inserted near midpoint of dorsal edge.

**Thorax:** Scutum green to green-blue with moderately dense silver-gray pollen, with bronze to bronze-green stripes between acrostichal and dorsocentral setae and along intra-alar setae; posterior slope of scutum and scutellum blue-green with some silver pollen; notopleuron and postpronotum (humerus) covered with dense silver pollen, usually with some blue-green or bronze reflections; 6 dorsocentral setae; 6–9 large acrostichal setae (largest two-thirds size of dorsocentral setae), in a single row; 2 notopleural setae; postpronotum with 1–3 strong, black setae and often a few smaller white hairs; 2 presutural intra-alar setae (one near suture); 1 presutural and 2 postsutural supra-alar setae; 1 postalar seta; scutellum with 6 large marginal setae (3 per side), no additional hairs; proepisternum with 1 dorsal and 1 ventral tuft of white hairs. Pleura metallic green-bronze, covered with dense silver-gray pollen, without setae or hairs.

**Legs:** Legs concolorous with pleura, but with slightly less silver-gray pollen, femoral ‘knees’ very narrowly orange. Coxa I uniformly covered with white hairs on anterior surface (length of hairs subequal to width of coxa I), with a couple black, slender setae near apex. Coxa II with scattered white hairs anteriorly, a few white and black setae near apex, and a black *ad* seta just beyond 1/2. Coxa III with a few short, white hairs on anterior surface and a black dorsal seta near 1/2. Femur I with row of short, black, *pd* setae on apical half (length ≤ half width of femur) and scattered, short, white hairs near base. Femur II with row of posterior hairs/setae (length ≤ width of femur), those on basal half white (longest near to just beyond middle of femur) and becoming black and shorter apically. Femur III with white hairs (length subequal to width of femur) dorsally to posteriorly at base. Tarsus I(2) very short, about as long as wide, with ventral row of setae/setulae (Fig. 3D). Ratios of tibia:tarsomeres for leg I: 16-8-2-4-3-2; for leg II: 26-20-8-4-2-2; for leg III: 37-20-17-6-2-2.

**Wing** (Fig. 5C): Hyaline, infuscated on anterior third, especially between R_2+3_ and R_4+5_ (mostly along veins apically); with a longitudinal spurious vein between R_4+5_ and M_1_ that is arched anteriorly near 2/3 of wing and terminates near midpoint of a semicircular, translucent, area that is white in certain lights and enclosed within a small, brown cloud; with a diffuse brown spot just above spurious vein at arch and small brown spot on CuA_1_ near dm-cu. Apex of wing shallowly excavated at M_1_, with a broad, blunt, apically-pointed lobe between M_1_ and CuA_1_, this lobe without setae. Calypter yellow with a fan of long, pale yellow setae at apex. Halter pale yellow.

**Abdomen:** Cylindrical, elongate, blunt at apex (Fig. 2C); T1 metallic green with dense silver pollen laterally becoming somewhat less dense dorsally. T2-T4 blue-green with dense silver pollen basally, bronze apically. T5 dark bronze with metallic green reflections and sparse silver pollen. T6 dark bronze with blue-green reflections and with dense silver pollen. T1-T3 with yellowish hair laterally, longest on T1 and T2, without black hairs or setae. Sternites bronze with dense silver-gray pollen. S1 bare except for lateral small tuft of yellow hairs at extreme base. S2 and S3 with sparse yellow hairs. S4 mostly bare. Hypopygium (Fig. 10C): cercus very long (as long as abdomen in unshriveled specimens), slender, cylindrical, with yellow hairs that are longest medially (Fig. 2C).

**Female.** Body length 7.0–8.0 mm, wing length 6.5–7.5 mm. Similar to male except for face twice as wide, dark green to violet obscured by moderate to dense brown pollen; palpus larger, black with silver pollen apically, with dense golden-brown pollen in middle at base, and black setae. Acrostichal setae large, stout, two-thirds to three-quarters size of dorsocentral setae. Femur I with *pd* row of black setae on apical half (longest subequal to half width of femur) and sparse, very short white hairs on posterior and ventral surface. Femur II posteriorly with row of white hairs/setae on basal half to two-thirds (longest at base and just beyond middle) and black setae on apical half to one-third (longest of these posterior hairs/setae subequal to width of femur). Wing (Fig. 16D) hyaline, with three to four diffuse brown clouds: one in cells r_4+5_ and bm+dm near midpoint of wing (this often appears like two ‘spots’ but actually connected by sometimes light brown clouding), one light brown cloud on M_1_ beyond crossvein dm-cu, and usually with very small light brown clouding on R_4+5_ at apex of wing; sometimes a small brown cloud is evident on CuA_1_.

#### Remarks.

The type specimens were collected by Van Duzee (1917: 128) "at Berkeley, California, May 8th, on a wall of rock in a little canyon; the wall was covered with water-soaked moss". Specimens have commonly been collected on or near beaches and sea cliffs.

Copulation of this species was described by Peter Dyte (in litt. to Richard Hurley, 1988): "A pair of *Liancalus limbatus* V.D. were seen in copula on a seepage at Wreck Beach, Vancouver, B.C., Canada, on 5.vii.1988, at 16.18 hrs. They were captured by placing a 3×1 in[ch] tube over them and they remained in copula in the tube until 17.20 hrs...All her tarsi and his mid and hind tarsi were on the substrate, but his foretarsi were held against the sides of her third abdominal segment with their apices projecting slightly below her abdomen. Thus the modified second segment of his foretarsus would have been touching her abdomen, though the separate tarsal segments could not be distinguished with the hand lens available. His long genital lamellae pointed forwards below the female abdomen but not touching it".

#### Distribution.

This species occurs primarily along or near the Pacific coast from central California to Alaska, but also inland in Idaho, Oregon, Washington and British Columbia (Fig. 14B).

#### Type material examined.

**HOLOTYPE** ♂, labeled: "Berkeley, Cal./ May 8 1915/ MC Van Duzee"; "Liancalus/ limbatus/ Holotype. Van Duzee" (CAS). **PARATYPE** ♀, same data as holotype (CAS).

#### Additional material examined.

**CANADA.** **British Columbia:** Vancouver, 8.viii.1917, ALM (1 ♂, USNM), P Rupert, 11.ix.1921, W.B. Anderson (2 ♂, CNC), 27 mi. E Pr. Rupert, on tide flats, 24.vi.1960, R.J. Pilfrey (1 ♀, CNC), Vancouver, Point Grey, on seepage on earth cliff, 23.vii.1973, JRV (4 ♂, 4 ♀, CNC), same as previous, 26.vii.1973 (6 ♀, CNC), same as previous, 23.ii.1973 (1 ♀, CNC), same as previous, 15.v.1973 (1 ♀, CNC), Robson, 19.viii.1947, H.R. Foxlee (1 ♀, CNC), same as previous, 3.ix.1948 (1 ♀, CNC), same as previous, 21.x.1955 (1 ♂, UBCZ), Cultus Lake, 8.vii.1948, H.R. Foxlee (1 ♀, CNC), Vancouver, 28.iii.1902, C.W. Johnson (1 ♀, MCZ), Hwy 97, Bijoux Falls Park, mileage 115.3 miles (185.6 km) from Prince George, Stop-234, 6.viii.1989, PHA (1 ♀, CAS), Vancouver, University of British Columbia, Wreck Beach Trail, behind beach, 8.vii.1988, PHA (1 ♀, CAS). **USA.** **Alaska:** Juneau Co., Thane, 29.vii.1958 (1 ♂, WSU). **California:** Santa Cruz Mts., 24.vii.1895 (1 ♂, 1 ♀, LACM); Humboldt Co., Arcata, 19.v.1976, M. Terzich (1 ♀, MTEC), Arcata, 22.iii.1981, RLH (2 ♀, MTEC); Marin Co., Muir Woods, 7.viii.1915, ALM (1 ♂, 1 ♀, USNM), Ross, 22.vi.1955, H.L. Mathis (1 ♂, 1 ♀, UCDC), Stinson Beach SP, 2nd inlet S main beach, sweeping rocks and vegetation about waterfall at ocean beach, 21.vi.1970, I.A. Boussy (1 ♂, 1 ♀, CAS), Mt Tamalpais, 26.v.1974, D.G. Denning (1 ♂, UCDC), Point Reyes, 19.iv.1980, S.A. Marshall (2 ♂, 2 ♀, DEBU), Phoenix Lake Pk., 22.v.1949, C.H. Spitzer (1 ♀, EMEC), same as previous, 30.v.1949 (1 ♂, EMEC); Monterey Co., Pacific Grove, 5-8.v.1906, JMA (8 ♂, 8 ♀, USNM); Napa Co., Butts Cyn, 0.5 mi. S Napa Co. line, 7.v.1970, E.E. Grissell & R.F. Denno (1 ♀, UCDC); San Mateo Co., Martin’s Beach, 9.ix.1969, T.W. Davies (1 ♂, 2 ♀, CAS), Memorial Park, 15.vii.1951, PHA (1 ♀, CSCA); Santa Barbara Co., Sta. Barbara, 6.vii.1917, JMA (1 ♂, 4 ♀, USNM); Santa Cruz Co., Capitola, 7-12.vi.1940, M.T. & H.B. James (14 ♂, 14 ♀, CSUC, WSU), Davenport, 1.ix.1948, W.W. Wirth (3 ♂, 1 ♀, EMEC, USNM), Santa Cruz, beach, 15-17.vi.1950, M.T. James (5 ♂, 11 ♀, WSU), 1 mi. N Davenport, on beach, 4.vii.1967, A. & A. Gillogly (1 ♂, UCR), Santa Cruz, beach, 15-17.vi.1950, M.T. James (4 ♂, 12 ♀, WSU), Santa Cruz, 9.vii.1972, D.G. Denning (1 ♀, UCDC); Sonoma Co., Bodega Bay, 13.v.1963, C.G. Moore (1 ♂, UCDC), Stillwater Cove, 23.v.1954, E.I. Schlinger (2 ♂, 2 ♀, UCDC), same as previous, J.G. Downey (1 ♂, 1 ♀, UCDC). **Idaho:** Adams Co., Little Salmon R, 18 mi. N New Meadows, 8.viii.1979, RLH (1 ♀, MTEC); Boise Co., Canyon Crk, 30 mi. NE Lowman, 15.ix.1981, RLH (1 ♀, MTEC); Kootenai Co., Beauty Crk, near Lake CD’A, 31.viii.1976, D.F. Veirs (1 ♀, WFBM); Latah Co., Mts Moscow, 25.vii.1920, R.C. Shannon (1 ♂, 1 ♀, USNM), Moscow Mts, East Twin Peak, 19.vii.1983, R.S. Zack (2 ♂, 1 ♀, WSU), Lower Sand Crk, nr. Bonami Crk, 16 mi. E Potlatch, 2900 ft, 5.viii.1979, WJT (3 ♂, WSU), same as previous, 9.viii.1979 (2 ♂, 6 ♀, WSU), same as previous, 28.viii.1982 (1 ♂, 1 ♀, WSU), 7 mi. N Troy, near Big Meadow Recreation Area, 3000 ft, 31.vii.1979, WJT (1 ♂, 1 ♀, WSU), Big Meadow Recreation Area, 7 mi. NNE Troy, 3000 ft, 7.viii.1986, WJT (1 ♂, 1 ♀, WSU); Nez Perce Co., Juliaetta Falls, 5.2 mi. S. Juliaetta, 3.iii.1983, J. Jenkins (2 ♀, MTEC). **Oregon:** Mt Hood, 29.vii.1966, FCH (10 ♂, 4 ♀, EMUS, FSCA, MTEC); Benton Co., Mary’s Peak, Parker Crk, roadside seepage, 26.ix.1967, KJG (1 ♂, ODAC), Marys Peak, Hwy 34, 4097', 16.vii.1968, B.V. Peterson (1 ♂, 1 ♀, CNC), Parker Crk, Marys Peak Rd, 1.vii.1971, G. Steyskal (1 ♂, 1 ♀, OSAC), Mary’s Peak nr Corvallis, F.R. Cole (1 ♂, EMEC); Coos Co., Charleston, marine biological station, 9.vii.1954, M.T. James (1 ♀, WSU); Hood River Co., Hood River, seepage over road cut, 15.viii.1966, KJG (2 ♂, 1 ♀, CAS, OSAC), 8 mi. NW Mt Hood, Lolo Pass Rd, roadside seepage, 9.x.1966, KJG (1 ♂, OSAC), 20 mi. S Hood River, vertical roadside seepage,16.viii.1966, KJG (1 ♂, 2 ♀, CAS, MTEC, OSAC), Hood River, 21.vi.1917, F.R. Cole (1 ♀, CAS); Jackson Co., 10 mi. S Ruch, stream margins, 22.v.1964, KJG (2 ♀, ODAC); Josephine Co., Grants Pass, black light trap, 15.ix.1965, KJG (1 ♂, ODAC); Lincoln Co., Agate Beach, base sea cliff, 16.v.1976, R.L. Westcott (1 ♀, ODAC), 15 mi. S Newport, seepage on sea cliffs, 13.vii.1966, KJG (1 ♂, 3 ♀, FSCA, OSAC), Newport, seeps along coastal cliffs, 22.vii.1983, WJT (1 ♂, 10 ♀, WSU), 20 mi. S Hood River, vertical roadcut seepage, 16.viii.1966, KJG (1 ♂, OSAC), Yachats, 20.vi.1939, G.E. Bohart (1 ♂,UCDC), 1 mi. N Depoe Bay, H_2_O spring above beach, 24.vi.1956, J.D. Lattin (1 ♂, OSAC); Linn Co., 1 mi. S Marion Forks, 15.v.1967, KJG (2 ♀, ODAC), same as previous, seepage along road cut, 31.vii.1966 (5 ♂, 2 ♀, CAS,CSCA, ODAC, OSAC), same as previous, shaded vertical seepage, 24.viii.1967 (7 ♂, 4 ♀, ODAC, OSAC, MTEC), same are previous, 21.vii.1969, E.M. Fisher (1 ♀, CSCA); Multnomah Co., Larch Mt, 15.viii.1966, KJG (2 ♂, FSCA), Portland, 16.vi.1926, A. Spuler (1 ♂, SEMC), Troutdale, 1.viii.1965, FCH (2 ♂, 2 ♀, FSCA, MTEC), same as previous, 23.vi.1963, L.S. Miller (1 ♀, FSCA); Tillamook Co., Oceanside, seepage along ocean cliff, 29.vii.1966, KJG (2 ♀, ODAC). **Washington:** "Washington (state)" (♀ paralectotype of *Liancalus similis*, SEMC), Mt Adams, 24.vii.1921, ALM (1 ♀, USNM); Asotin Co., 17 mi. S Anatone, nr. Grande Ronde River, 1950 ft, dripping spring, 10.viii.1976, WJT (1 ♂, WSU); Columbia Co., Tucanon RS, Blue Mts, 13.viii.1922, V.N. Argo (1 ♂, 2 ♀, USNM); King Co., Lake Washington, 12.viii.1917, ALM (7 ♂, 6 ♀, USNM); Okanogan Co., 9 mi. W Conconully, Salmon Mdws, 4500 ft, 3-6.vii.1975, N.E. Woodley (1 ♀, WSU), Seattle (1 ♂, 2 ♀, OSAC); Pacific Co., Ilwaco, vii.1917, ALM (3 ♀, USNM); San Juan Co., Friday Harbor, 23.vii.1905, JMA (2 ♂, 2 ♀, USNM), same as previous, 27.vii.1905 (2 ♂, USNM), Olga, 17.v.1910 (2 ♀, USNM); Skagit Co., 2 mi. S Sedro-Woolley, 28.viii.1971, H.P. Stene (2 ♂, WSU); Stevens Co., Deer Lake, near Chewelah, 6-7.v.1972, M.T. James (1 ♀, WSU); Whatcom Co., 9 mi. N Concrete, 15.vii.1969, R. Turnbow (1 ♀, UGCA).

### Liancalus pterodactyl

Taxon classificationAnimaliaDipteraDolichopodidae

Runyon & Hurley sp. n.

http://zoobank.org/2C4A491C-2092-404D-B9E6-DEDB36EE233B

Figs 1 , 2D , 3A , 4A , 7 , 11A , 13 , 16A

#### Diagnosis.

Males are most similar to *Liancalus hydrophilus* but have a longer, more slender setae-bearing lobe on posteroapical wing margin (Figs 4, 7). Cerci of male *Liancalus pterodactyl* have long setae confined mostly to apical half (Fig. 2D), whereas *Liancalus hydrophilus* has long, evenly-spaced setae along the full-length of the cerci (Fig. 8). Females are distinguished by the dorsal one-quarter or more postcranial hairs dark brown to black (other Nearctic species have at most a few dark hairs).

#### Description.

**Male.** Body length 9.0–12.0 mm, wing length 7.5–8.5 mm. **Head:** Face nearly parallel-sided on dorsal half, widening toward clypeus; frontoclypeal suture near mid-face, distinct; eyes with short hairs between facets; face green-bronze (more green immediately below antenna) with silver pollen most noticeable along eyes and on ventral half of face; ommatidia essentially the same size throughout. Vertex concolorous with face, usually with dense silver pollen. Vertical setae on small elevation; ocellar tubercle prominent with 2 large setae (subequal in size to vertical setae); with 2 postocellar setae which are two-thirds length ocellar setae; postocular setae approximately half length of vertical setae with dorsal two-thirds of postocular setae black (approximately 20 black setae), ventral third (approximately 10 setae) white and more slender and slightly longer than other postocular setae. Ventral postcranial hairs (beard) abundant, white with dorsal third to one-quarter black. Palpus black, covered with silver pollen and sparse black hairs. Antenna (Fig. 1) black, first flagellomere about as long as wide, rounded apically, arista inserted near midpoint of dorsal edge.

**Thorax:** Scutum blue-green with bronze stripes between acrostichal setae and dorsocentral setae, and along intra-alar setae; scutellum and posterior slope of scutum blue-green, posterior slope of scutum sometimes with bronze medially; notopleuron and postpronotum (humerus) covered with dense silver pollen, usually with some blue-green reflections; usually 6–10 dorsocentral setae; 8–14 long acrostichal setae (≥ 2/3 length of dorsocentral setae), in a single row; 2 notopleural setae; postpronotum with 1–2 strong setae and often a few smaller hairs or setae; 2 presutural intra-alar setae; 1 presutural and 2 postsutural supra-alar setae; 1 postalar seta; scutellum blue-green with 8 (rarely 9) large marginal setae (4 per side), no additional hairs; proepisternum with 1 dorsal and 1 ventral tuft of white hairs. Pleura metallic green-bronze, covered with dense silver-gray pollen, without setae or hairs (Fig. 1).

**Legs:** Legs concolorous with pleura. Coxa I (Fig. 1) uniformly covered with white, slender hairs on anterior surface (length of hairs subequal to width of coxa I), with a few black, slender setae at apex. Coxa II with white hairs anteriorly, a couple black setae near apex, and a black *ad* seta just beyond 1/2. Coxa III with scattered white hairs and a black dorsal seta near 1/2. Femur I with sparse white hairs on ventral surface (length ≤ width of femur). Femur II with row of short (≤ width of femur) posterior to *pv* setae on distal half, those near middle of femur white, longest and becoming black and shorter apically; with a row of very short black *ad* setae preceding usual preapical seta. Femur III with scattered white hairs (length ≤ width of femur) on dorsal and posterior surface at base. Tarsus I(2) short (length subequal to width), slightly thickened, with ventral brush of setulae (Fig. 3A). Ratios of tibia:tarsomeres for leg I: 18-7-2-5-3-2; for leg II: 26-22-7-4-2-2; for leg III: 38-17-13-4-2-2.

**Wing** (Figs 4A, 7): Hyaline, with light brown clouding between R_2+3_ and R_4+5_ apically, most distinct along veins; with a longitudinal spurious vein immediately above M_1_ that ends near junction of M_1_ with dm-cu; apical part of membrane between R_4+5_ and M_1_ with a narrow, translucent area that is white in certain lights and enclosed within a small, brown cloud (Fig. 7). Fourth costal sector (between R_4+5_ and M_1_) flattened with a cluster of 3–4 long, black setae at apex of M_1_ that are usually fused apically; wing margin between M_1_ and CuA_1_ with a long (length > 3× width), slender, finger-like projection bearing several black setae at apex. Calypter yellow with a fan of long, pale yellow setae at apex. Halter pale yellow (Fig. 1).

**Abdomen:** Cylindrical, elongate (Fig. 2D); T1 metallic blue-green, covered with dense silver pollen. T2-T4 mostly metallic blue-green with silver pollen, with apical one-third bronze, usually bronze dorsally at base. T5 dark bronze with metallic green dorsally and sparse silver pollen. T6 dark metallic blue-green with sparse to moderate silver pollen. T1-T3 with long, white hair laterally, longest on T1 and T2. Sternites bronze with sparse silver-gray pollen. S1 bare except for lateral tuft of white hairs at extreme base. S2 and S3 with sparse long, white hairs. S4 mostly bare. Hypopygium (Fig. 11A): cerci as long or longer then abdomen, slender, cylindrical, with pale yellow setae on ventral surface that are most dense and longest on apical half (Fig. 2D).

**Female.** Body length 7.5–9.0 mm, wing length 7.0–7.5 mm. Similar to male except for face wider; palpus covered with golden brown pollen, with denser, black setae. Femur I with *pd* row of black setae/hairs and relatively long (subequal to width of femur), white hairs on ventral and posterior surface. Femur II with pale hair on posterior surface extending nearly to base; tarsus I(2) normal, not unusually short or thickened. Wing (Fig. 16A) hyaline, of normal shape; with diffuse brown clouding immediately above M_1_ before junction with dm-cu, this clouding often continuing posteriorly into cell bm+dm.

#### Etymology.

The epithet, a noun in apposition, is in reference to the large size of this species – reminiscent of the large pterosaurs from the Jurassic; and the Greek *pteron* "wing" + *daktylos* "finger" in reference to the finger-like lobe on the wing (Fig. 4A).

#### Remarks.

This is perhaps the largest species of Dolichopodidae, at least in terms of body length.

#### Distribution.

This species occurs in the Northern Rocky Mountains of U.S. and neighboring Canada; one male was collected in the Trinity Alps of northern California (Fig. 13).

#### Type material.

**HOLOTYPE** ♂, labelled: "MONTANA: Gallatin Co./ Grotto Falls 7000'/ 22 mi S Bozeman/ 19-VIII-2001/ J. B. Runyon"; "HOLOTYPE/ ♂ *Liancalus*/ *pterodactyl*/ Runyon & Hurley" [red label] (MCZ). **PARATYPES:** **CANADA.** **Alberta:** Lake Louise, 30.x.1923, Eric Hearle (1 ♂, CNC), Banff, 6.x.1926, Eric Hearle (1 ♂, CNC). British Columbia: Robson, 13.iii.1957, H.R. Foxlee (3 ♀, UBCZ), same as previous, 14.iii.1957 (1 ♀, UBCZ), same as previous, 18.iii.1957 (1 ♀, UBCZ), same as previous, 17.x.1961 (1 ♀, UBCZ). **USA.** **California:** Trinity Co., Siligo Mtn, 7000 ft, 11.viii.1967 (1 ♂, CAS). **Idaho:** Bonner Co., 14 mi. NW Samuels, 26.ix.1969, W.F. Barr (1 ♀, WSU). **Montana:** Flathead Co., seep on Going to the Sun Rd nr Haystack Crk, Glacier NP, 22.ix.2003, J. Giersch (1 ♂, MTEC), Cattle Queen Crk at Highline Trail, Glacier NP, 48.832221, -113.799051, 1845 m elev, 11.ix.2012, J. Giersch (1 ♂, MTEC); Gallatin Co., Bozeman, 3.ix.1960, S. Wiegand (1 ♂, MTEC), Silken Skein Falls, 3 mi. S Hyalite Rsvr., 8333 ft, 11.viii.2000, JBR (1 ♂, MTEC), Palisade Falls, 1 mi. S Hyalite Rsvr., 7685 ft, 12.viii.2000, RLH & JBR (1 ♂, 2 ♀, MTEC), same as previous, 19.x.2000, JBR (2 ♂, MTEC), same as previous, 20.iv.2002, JBR (1 ♀, MTEC), same as previous, 20.viii.2009, JBR (3 ♂, 1 ♀, MTEC), same as previous, 12.x.2012, JBR (1 ♂, MTEC); Glacier Co., Cracker Lake inlet far West stream, Glacier NP, 48.742317, -113.651581, 1845 m elev, 9.ix.2011, J. Giersch (1 ♂, MTEC).

### Liancalus querulus

Taxon classificationAnimaliaDipteraDolichopodidae

Osten Sacken, 1877

**Figure 15.**
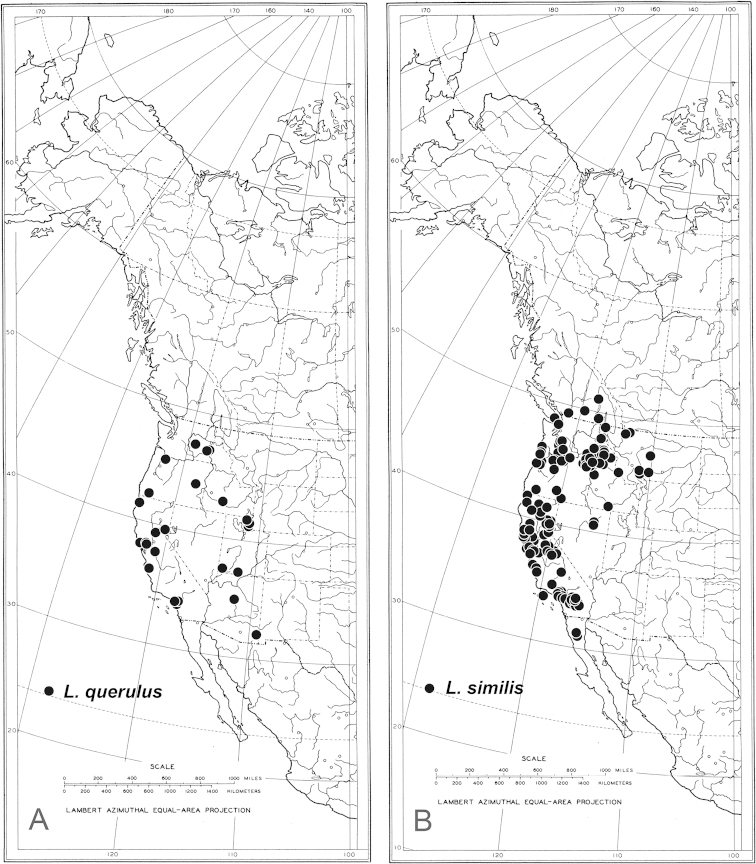
Known distributions of **A** *Liancalus querulus* Osten Sacken and **B** *Liancalus similis* Aldrich.

**Figure 16.**
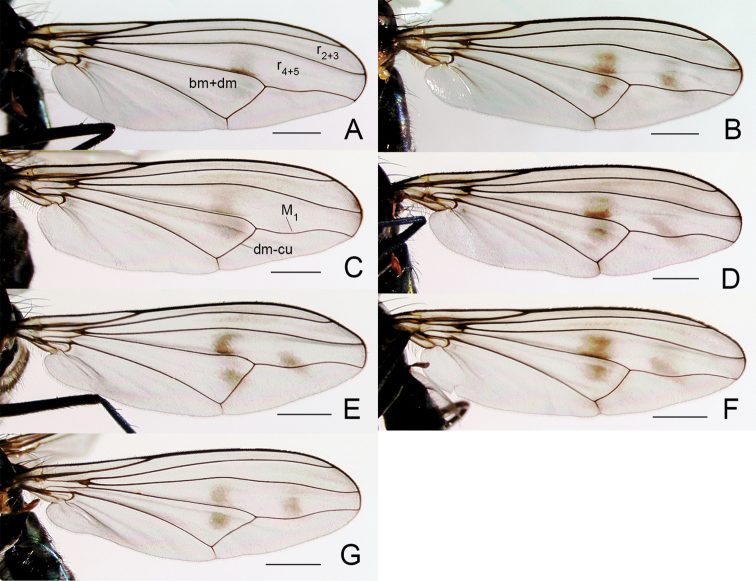
Wings of female species of *Liancalus*, **A** *Liancalus pterodactyl* sp. n. **B** *Liancalus genualis* Loew **C** *Liancalus hydrophilus* Aldrich **D** *Liancalus limbatus* Van Duzee **E** *Liancalus similis* Aldrich **F** *Liancalus sonorus* sp. n. **G** *Liancalus querulus* Osten Sacken. Wing cells bm+dm, r_2+3_ and r_4+5_ are labeled in (**A**); wing veins dm-cu and M_1_ are labeled in (**B**).

Figs 2F , 3F , 6A , 12A , 15A , 16G

#### Diagnosis.

This species is most similar to *Liancalus similis*: males of both have tarsus I with tarsomere 2 long (Fig. 3F–G), wings with apical brown clouding (Fig. 6), and very short cerci (Fig. 2E–F), but *Liancalus querulus* males have a smaller, semicircular brown cloud near wing apex and no black speck in cell m (Fig. 6A). Female wing with three distinct brown spots and with crossvein dm-cu meeting M_1_ at nearly 45° angle (Fig. 16G).

#### Redescription.

**Male.** Body length 6.5–8.0 mm, wing length 6.0–7.5 mm. **Head:** Face rather broad, uniformly covered with dense silver pollen. Ommatidia near face slightly larger than remaining ommatidia. Vertex covered with dense silver pollen, often with some metallic green or blue color near middle. Vertical setae on very small elevation; ocellar tubercle prominent with 2 large setae (subequal in size to vertical setae); with 2 postocellar setae which are three-quarters length ocellar setae; postocular setae slightly less than one-half length of vertical setae with dorsal one-third of postocular setae black (approximately 10 black setae), ventral two-thirds (approximately 15 setae) white and more slender and slightly longer than black postocular setae. Ventral postcranial hairs (beard) rather sparse, wholly white. Palpus black, covered with sparse to moderately dense silver pollen and sparse black hairs. Antenna black, first flagellomere about as long as wide, broadly pointed apically, arista inserted just beyond midpoint of dorsal edge.

**Thorax:** Scutum green to green-blue with sparse to moderately dense silver-gray pollen, with bronze stripes between acrostichal and dorsocentral setae, and along intra-alar setae; scutellum and posterior slope of scutum green-blue with sparse silver pollen; notopleuron covered with silver pollen and postpronotum covered with sparse brown-gray pollen, both with some blue-green reflections; corner of postpronotum above anterior spiracle narrowly orange; 6 dorsocentral setae; 4–11 small acrostichal setae (≤ 1/2 length of dorsocentral setae), in a single row; 2 notopleural setae; postpronotum with 1–2 strong, black setae and often a few smaller white hairs; 2 presutural intra-alar setae (one near suture); 1 presutural and 2 postsutural supra-alar setae; 1 postalar seta; scutellum with 6 large marginal setae (3 per side), no additional hairs; proepisternum with 1 dorsal and 1 ventral tuft of white hairs. Pleura metallic green-bronze, covered with dense silver-gray pollen, without setae or hairs.

**Legs:** Legs concolorous with pleura, but less silver-gray pollen, femoral ‘knees’ narrowly orange. Coxa I uniformly covered with white hairs on anterior surface (length of hairs subequal to width of coxa I), with a few black, slender setae at apex. Coxa II with a few white hairs anteriorly, a couple white setae near apex, and a black *ad* seta just beyond 1/2. Coxa III with a few white hairs and a black dorsal seta near 1/2. Femur I and II with short, white hairs posteriorly to *pv* on basal half (length < half width of femur). Femur III with some short white hairs (length ≤ width of femur) on dorsal and posterior surface at base. Tarsus I(1) short, approximately one-third length of tarsus I(2) (Fig. 3F); tarsus I(2) long, flattened ventrally and covered with dense setulae full-length; tarsus I(3) slightly thickened near or just beyond middle (Fig. 3F); tarsus I(3–4) with black, felt-like setulae on *pv* surface. Ratios of tibia:tarsomeres for leg I: 11-3-8-3-2-2; for leg II: 23-22-11-3-2-2; for leg III: 27-14-16-4-2-2.

**Wing** (Fig. 6A): Hyaline, with semicircular brown cloud near apex (clouding very faint in a few specimens). Wing relatively slender, rounded at apex, without outstanding hairs or setae. Veins R_4+5_ and M_1_ closely approximated and nearly parallel at apex of wing. Calypter yellow with a fan of long, pale yellow setae at apex. Halter pale yellow.

**Abdomen:** Cylindrical, elongate, slightly enlarged and rather blunt at apex (Fig. 2F); T1 metallic green-blue, with dense silver pollen laterally becoming less dense dorsally, with bronze along posterior edge and usually a narrow bronze stripe dorsally. T2-T4 bronze with large lateral blue-green spots covered with dense silver pollen. T5 dark bronze with metallic green reflections and sparse silver pollen. T6 bronze with blue-green reflections and with moderately dense silver pollen. T1-T3 with white hair laterally, longest on T1 and T2. Sternites bronze with silver-gray pollen. S1 bare except for lateral small tuft of white hairs at extreme base. S2 and S3 with sparse white hairs. S4 mostly bare. Hypopygium (Fig. 12A): cerci small, lacking long tubular filaments of other species, but with minute papilla where filaments originate in other species.

**Female.** Body length 5.0–7.5 mm, wing length 5.5–7.0 mm. Similar to male except for face slightly wider, dark blue-green covered with moderate golden-brown pollen that is most dense along eyes; palpus black with sparse silver pollen apically, dense golden-brown pollen in middle basally, and black setae. Femur II usually with posterior row of short (length less than half width of femur) white hairs on basal half, these hairs becoming black on apical half. Tarsus I(1) normal, about twice as long as tarsus I(2). Wing (Fig. 16G) hyaline, with three brown spots: one between R_4+5_ and M_1_ near midpoint of wing, one apically in cell bm+dm but not reaching crossvein dm-cu, and a spot on or just above M_1_ beyond crossvein dm-cu. Crossvein dm-cu meeting M_1_ at a nearly 45° angle.

#### Remarks.

Harmston and Knowlton (1945) found *Liancalus querulus* "in large numbers during late summer about masonry dams in the canyons near Logan and Ogden, Utah".

#### Distribution.

This species is rather uncommon, but widely distributed in the western U.S. (Fig. 15).

#### Type material examined.

**LECTOTYPE** (designated here to fix identity of the species) ♂, labelled: "Type/ 870" [red label]; "The Geysers, Cal./ May 5-7./ R. Osten Sacken."; "O. Sacken./ West. Dipt."; "Liancalus querulus O.S." [hand-written]; "MCZ-ENT/ 00302771" [with barcode]; "LECTOTYPE ♂/ Liancalus querulus/ Osten Sacken/ des. Runyon & Hurley" [red label] (MCZ). **PARALECTOTYPE:** Same data as lectotype, MCZ-ENT/ 00000870 (1 ♀, MCZ).

#### Additional material examined.

**USA. Arizona:** Cochise Co., Portal, S.W. Res. Sta. AMNH, 23.vi.1968, V.D. Roth & FCH (2 ♂, 2 ♀, MTEC); Coconino Co., Oak Crk Cyn, 25.vi.1986, J. Jenkins (3 ♂, MTEC). **California:** Contra Costa Co., Jewel L, 11.v.1948, W.W. Wirth (1 ♀, EMEC), Danville, 23.vi.1951, by small waterfall, F.X. Williams (2 ♂, CAS), same as previous, 3.vii.1952, by rapids at creek (1 ♂, CAS), same as previous, 24.vi.1952, by rapids (1 ♂, CAS); Humboldt Co., Orick, 3.vii.1950, L.W. Quate (1 ♀, EMEC); Los Angeles Co., Monrovia Cyn, 18.v.1966, J.C. Hall (1 ♂, UCR), Topanga Cyn, 10.v.1953 (1 ♀, LACM), Big Dalton Cyn, 19-23.vii.1952, S. Miyagawa (22 ♂, 8 ♀, CUIC, UCDC), same as previous, 23.vii.1952, H.L. Mathis (2 ♂, 2 ♀, UCDC), same as previous, 14-23.vii.1952, A.T. McClay (2 ♂, 3 ♀, UCDC), same as previous, 19.vii.1952, A.A. Grigarick (3 ♂, 1 ♀, UCDC), Tanbark Flat, 15.vi.1956, R.C. Bechtel (1 ♂, UCDC); Marin Co., Phoenix Lake Pk., 22.v.1949, C.H. Spitzer (1 ♂, 1 ♀, EMEC), same as previous, 30.v.1949 (1 ♂, EMEC); Monterey Co., Big Creek, 4.vi.1982, J. Powell (1 ♂, EMEC); Sierra Co., Hwy 49, creek at Rosassco Ravine, 2.4 km W Downieville, 890 m, 4.vii.1975, PHA (1 ♂, CAS); Stanislaus Co., Del Puerto Cyn, Frank Raines Park, 335m, 17.v.1970, PHA (1 ♂, CAS); Sutter Co., Sutter Buttes, 5.v.1940, G.E. Bohart (3 ♂, 2 ♀, UCDC). **Idaho:** Gooding Co., Hagerman, Thousand Springs, JMA (1 ♂, USNM). **Oregon:** Hood River Co., Hood River, 1.x.1917, F.R. Cole (1 ♂, CAS); Jackson Co. Beaver Sulfur Campground, 10 mi. S Ruch, resting on rock in stream, 28.vii.1967, KJG (1 ♂, 1 ♀, MTEC); Malheur Co., 9 mi. E Juntura, runoff from hot spring, 16.vi.1964, KJG (1 ♂, ODAC). **Utah:** Cache Co., Logan Meadows, 15.x.1939, G.F. Knowlton & FCH (3 ♂, CAS), Logan Cyn, 15.viii.1940, FCH (1 ♂, 2 ♀, MTEC); Kane Co., Grand Staircase-Escalante National Monument, Sheep Crk jct., Skutumpah Rd., 5531 ft, 37°29'43N 112°03'59W, 29.ix.1999, R.W. Baumann, K.T. Huntzinger, C.R. Nelson (1 ♂, BYU); Rich Co., Lakota, 5.vii.1957, FCH (3 ♂, 2 ♀, MTEC); Washington Co., Leeds, 27.iv.1940, G.F. Knowlton & FCH (2 ♂, CMNH, EMUS), same as previous, 9.vi.1940 (1 ♂, MTEC), Leeds Cyn, 13-16.ix.1984, Hansen & Youssef (1 ♂, 2 ♀, EMUS), same as previous, 15-18.vii.1980, malaise trap (1 ♂, EMUS), same as previous, 21.viii-4.ix.1985, W.J. Hansen (1 ♀, EMUS), same as previous, 29.vii.1965 (1 ♂, EMUS); Weber Co., Ogden Cyn, 10.x.1939, G.F. Knowlton & FCH (7 ♂, 4 ♀, CNC, CUIC, EMUS, MTEC, OSU), same as previous, 9.x.1937 (1 ♂, EMUS). **Washington:** Grant Co., O’Sullivan Dam, 25.x.1953, H.G. Davis (1 ♂, WSU); Whitman Co., Lyle Grove, near Pullman, malaise trap, 1.vii.1968, H.S. Telford (1 ♀, WSU), Steptoe Cyn, 10 mi. SW Colton, 3.viii.1974, WJT (1 ♀, WSU).

### Liancalus similis

Taxon classificationAnimaliaDipteraDolichopodidae

Aldrich, 1893

Figs 2E , 3G , 6B , 9 , 12B , 15B , 16E

#### Diagnosis.

Males are easily distinguished by having the apical third of wing mostly brown and with a slightly raised, black speck near middle of cell m (Fig. 6B). Female wing has three distinct brown spots, one of which intersects M_1_ beyond crossvein dm-cu, and with crossvein dm-cu meeting M_1_ at nearly 90° angle (Fig. 16E).

#### Redescription.

**Male.** Body length 6.5–8.0 mm, wing length 6.0–7.5 mm. Habitus (Fig. 9). **Head:** Face rather narrow, nearly parallel-sided, slightly widening toward clypeus, uniformly covered with dense silver pollen. Ommatidia near vertex slightly smaller than remaining ommatidia. Vertex covered with dense silver pollen, often with some metallic blue-green color visible near middle. Vertical setae on small elevation; ocellar tubercle prominent with 2 large setae (subequal in size to vertical setae); with 2 postocellar setae which are two-thirds length ocellar setae; postocular setae approximately one-third size of vertical setae with dorsal one-third of postocular setae black (approximately 10 black setae), ventral two-thirds white (approximately 12 setae) and more slender and slightly longer than black postocular setae. Ventral postcranial hairs (beard) rather sparse, wholly white. Palpus black, covered with dense silver pollen and sparse white hairs. Antenna black, first flagellomere about as long as wide, broadly pointed apically, arista inserted near midpoint of dorsal edge.

**Thorax:** Scutum blue-green obscured with dense silver-gray pollen, with bronze stripes between acrostichal and dorsocentral setae, and along intra-alar setae; scutellum and posterior slope of scutum green-blue with sparse gray or brown pollen; notopleuron and postpronotum (humerus) covered with dense silver pollen, usually with some blue-green reflections; usually 6 dorsocentral setae; 2–6 short acrostichal setae (≤ 1/2 length of dorsocentral setae), in a single row; 2 notopleural setae; postpronotum with 1–2 strong, black setae and often a few smaller white hairs; 2 presutural intra-alar setae (one near suture); 1 presutural and 2 postsutural supra-alar setae; 1 postalar seta; scutellum with 6 large marginal setae (3 per side), no additional hairs; proepisternum with 1 dorsal and 1 ventral tuft of white hairs. Pleura metallic green-bronze, covered with dense silver-gray pollen, without setae or hairs.

**Legs:** Legs concolorous with pleura, but with less silver-gray pollen. Coxa I uniformly covered with white hairs on anterior surface (length of hairs subequal to width of coxa I); with a few black, slender setae at apex. Coxa II with a few white hairs anteriorly, a couple white setae near apex, and a black *ad* seta just beyond 1/2. Coxa III with a few white hairs and a black dorsal seta near 1/2. Femur I and II with some short, white hairs ventrally at base (length < half width of femur). Femur III with white hairs (length ≤ width of femur) on dorsal and posterior surface at base. Tarsus I(1) short, approximately one-quarter length of tarsus I(2) (Fig. 3G); tarsus I(2) long, flattened ventrally with dense row of red-brown *av* setulae full-length and slightly longer row of *pv* setae/setulae. Ratios of tibia:tarsomeres for leg I: 14-2-8-3-3-3; for leg II: 29-20-11-3-2-2; for leg III: 33-14-15-4-2-2.

**Wing** (Fig. 6B): Hyaline, with extensive brown clouding on most of apical half; cell m (beyond crossvein bm-cu) with a minute, raised, black speck near middle – the area surrounding this speck usually lacks brown clouding. Veins R_4+5_ and M_1_ widely separated and parallel to slightly diverging at apex of wing. Wing broadly and rather evenly rounded apically, without outstanding hairs or setae. Calypter yellow with a fan of long, pale yellow setae at apex. Halter pale yellow.

**Abdomen:** Cylindrical, elongate (Fig. 2E); T1 metallic blue-green, with dense silver pollen laterally, sparser pollen dorsally and often with diffuse narrow bronze stripe. T2-T4 with large lateral blue-green spots covered with dense silver pollen, with apical one-third to one-half bronze, narrowly bronze dorsally. T5 dark bronze with metallic green reflections and sparse silver pollen. T6 bronze with some blue-green reflections and with dense silver pollen. T1-T3 with white hair laterally, longest on T1 and T2. Sternites bronze with dense silver-gray pollen on S1 and S2, sparser pollen apically. S1 bare except for lateral small tuft of white hairs at extreme base. S2 and S3 with sparse white hairs. S4 mostly bare. Hypopygium (Fig. 12B): cerci broad basally with very short slender, cylindrical lobe (subequal in length to first flagellomere of antenna), with small white hairs.

**Female.** Body length 5.0–7.5 mm, wing length 5.5–7.0 mm. Similar to male except for face wider, dark blue-green covered with moderate golden-brown pollen; palpus dark brown-black with sparse golden-brown pollen and black setae. Femur II with row of short (length less than half width of femur) hairs full-length posteriorly to *pv*, those on basal half white, those on apical half black. Tarsus I(1) normal, distinctly longer than tarsus I(2). Wing (Fig. 16E) hyaline, with three distinct brown spots: one between R_4+5_ and M_1_ just beyond midpoint of wing, one near apex of cell bm+dm and just crossing crossvein dm-cu, and a spot on M_1_ beyond crossvein dm-cu. Cell m without black speck near middle. Crossvein dm-cu meeting M_1_ at nearly 90°.

#### Remarks.

Aldrich (1893) states the type specimens are from "Washington (state)" and described *Liancalus similis* from one male and two females, and noted that "the wings of one [female] specimen have more brown, which takes the form of three well defined spots, but this is evidently variable". The female specimen with three well defined spots is *Liancalus similis*, but the other female is a specimen of *Liancalus limbatus* which co-occurs with *Liancalus similis* in Washington State.

This species is common at Palisade Falls in Gallatin County, Montana where the senior author (JBR) has observed females feeding (Suppl. material 1) and males interacting (Suppl. materials 2–3). Suppl. material 3 shows the typical looping flight of *Liancalus* species.

The immature stages of *Liancalus similis* were described by Corpus (1986). Mouthparts of *Liancalus similis* were described and illustrated by Cregan (1941).

#### Distribution.

This species is common from southern California north through the Cascades and east to the Northern Rockies, but generally absent from the Great Basin (Fig. 15B).

#### Type material examined.

**LECTOTYPE** (designated here to fix identity of the species) ♂, labelled: “W.J." [hand-written]; "COTYPE/ Liancalus/ similis/ Ald." [red label, one wing on pin below this label]; "LECTOTYPE/ Liancalus/ similis Aldrich/ des. Runyon & Hurley 2014" [red label] (SEMC). **PARALECTOTYPE** ♀, same data as lectotype (SEMC).

#### Additional material examined.

**CANADA.** **British Columbia:** 13 km N Revelstoke-Hwy 23, cascading stream over roadcut, 2.vii.1989, B.J. Sinclair (1 ♂, 3 ♀, CNC), Robson, H.R. Foxlee, numerous dates between 1955 and 1969 (2 ♂, 49 ♀, UBCZ), Harrison Mills, 16.vii.1953, W.R.M. Mason (1 ♀, CNC), Langford, 30.iii.1957, D. Evans (1 ♀, CNC), Summerland, Shingle Crk, 7.x.1932, A.N. Gartrell (1 ♀, CNC). **MEXICO.** **Baja California:** Sierra San Pedro Martir, 17 km. E Park entrance, 2900 m, 7.ix.1980, DDW (3 ♂, 1 ♀, CAS), San Pedro Martir, La Grulla, 12.vii.1953, PHA (1 ♀, CAS). **USA.** **California:** Yosemite NP, 1.viii.1940, D.E. Hardy (3 ♂, 2 ♀, MTEC, SEMC); Santa Cruz Mts., 24.vii.1895 (1 ♀, LACM), same as previous, 27.viii.1895 (1 ♂, LACM); Mt Saint Helena, 28.vii.1940, B. Brookman (1 ♂, CAS); Yosemite, 12.vi.1935, ALM (1 ♂, USNM); Alameda Co., Berkeley, 16.v.1951, R. Morgan (1 ♂, LACM); Amador Co., Forest Home, 26.viii.1944, ALM (2 ♂, 2 ♀, USNM); Contra Costa Co., Mt Diablo, 2.viii.1951, F.X. Williams (1 ♂, CAS), same as previous, 2000 ft, 23.viii.1951 (1 ♀, CAS), same as previous, wet wall of spring, 18.vii.1951 (1 ♀, CAS); Del Norte Co., 6 mi. N Gasquet, 11.i.1986, RLH (1 ♀, MTEC); El Dorado Co., Fred’s Place, 20.viii.1950, L.W. Quate (1 ♂, EMEC); Fresno Co., Glen Meadow Crk, above Dinkey Crk Ranger Station, 1755 m, 3.viii.1975, PHA (1 ♂, CAS), Bolsillo Crk at Bolsillo cmgrd, SW Mono Hot Springs, 2270 m, 8.viii.1975, PHA (1 ♂, 1 ♀, CAS); Humboldt Co., Crk 1/3 mi. W Ruby Crk above Willow Crk, 25.iv.1987, Baumann, Stark, Nelson &Wells (1 ♀, BYU); Kern Co., Mt Pinos, Iris Meadow, 2670m, malaise, 2-3.vi.1992, J. Skevington, A. Goering (1 ♂, DEBU); Lake Co., Kelseyville, 9.v.1953, W.H. Lange (1 ♀, UCDC); Los Angeles Co., Singing Sprs, 29.viii.1958, Menke & Stange (7 ♂, 9 ♀, LACM), Ranch - 2.5 mi. SSW Valyermo, 4800', 18.viii.1962, N. McFarland (2 ♂, OSAC), Angeles Nat. Forest, Windy Spr, 27.vi.1974, DDW (3 ♂, 8 ♀, CAS), July, Collection Coquillett (1 ♂, USNM), San Gabriel Mts, Cloudburst Cyn, T2N, R12W, Sec. 14, 4000 ft, 30.iv.1972, J.P. & K.E. Donahue (1 ♂, LACM), Big Dalton Cyn, 23.vii.1952, S. Miyagawa (1 ♀, LACM), Tanbark Flat, 12.vii.1952, S. Miyagawa (1 ♂, 3 ♀, FSCA, UCDC), same as previous, 24.vi.1952 (1 ♀, UCDC), same as previous, 5.vii.1952, R.L. Anderson (1 ♀, UCDC), same as previous, 16.vii.1952, D.E. Barcus (2 ♂, 2 ♀, UCDC), same as previous, 10.vii.1950, F.X. Williams (5 ♀, CAS), Monrovia Cyn, 18.v.1966, J.C. Hall (1 ♀, UCR), Tujunga Cyn, 12.vii.1962 (2 ♂, 1 ♀, MTEC), 0.5 mi. E Islip Saddle, Cortelyou Spr on Hwy 2, Angeles N.F., 25.iv.1977, DDW (2 ♀, CAS), N Fk. San Gabriel R, along Hwy 39, 2 mi. S Coldbrook Stn., 25.iv.1977, DDW (1 ♀, CAS), San Antonia Cyn, Ontario, 25.vii.1907 (12 ♂, 12 ♀, CAS, OSU), 1/4 mi. down Cooper Cyn Trail, near Mt Waterman Ski Area, 31.iii.1981, S.M. Clark (1 ♀, MTEC), 1 mi. NE Crystal Lake, 1.vii.1962, J.F. Lawrence (2 ♀, MCZ); Marin Co., Point Reyes National Seashore, 22.vi.1975, D.G. Denning (1 ♂, 1 ♀, UCDC), Mill Valley, Blithedale Ridge, Lee Street, 110 m, 27.iv.1965, PHA (1 ♀, CAS), Alpine Lake, 18.v.1958, D.C. Rentz (1 ♂, CAS), Mt Tamalpais, vicinity of Rock Sprs, ca. 610 m, 13.v.1978, PHA (1 ♂, CAS); Mariposa Co., Yosemite NP, Boundary Hill, Research Res. Area, 4.vii.1971, R.P. Allen (1 ♂, CSCA), seepage along Hwy 49 between Bear Valley and Coulterville, 3.v.1978, D.D.Wilder (1 ♂, 1 ♀, CAS), Summerdale Forest Camp on Big Crk, 1520m, 28.vi.1973, PHA (1 ♀, CAS); Monterey Co., Willow Crk, 7.viii.1962, E.I. Schlinger (1 ♀, UCR), Big Crk, 4.vi.1982, J. Powell (1 ♀, EMEC), Los Padres Nat. Forest, Escondido Cpgd, 22.v.1976, DDW (1 ♀, CAS); Nevada Co., Sagehen Crk, 19.vii.1978, R.B. Kimsey (3 ♂, UCDC), same as previous, L.D. French (3 ♀, UCDC), same as previous, 8.vii.1980, R.M. Bohart (1 ♀, UCDC); Plumas Co., Squirrel Crk, 8 mi. E Quincy, 3900', 15.v.1982, J.A. Powell (2 ♀, EMEC), Upper Jamison Crk, 3 mi. SW Johnsville, 5800', 27.vi.1989, RLH (1 ♂, 5 ♀, MTEC), Wolf Crk, 6 km NW Greenville, 1180 m, 15.viii.1977, PHA (1 ♂, 1 ♀, CAS); Riverside Co., Lake Hemet, 29.vii.1964, M.E. Irwin (1 ♂, 1 ♀, LACM), Fuller Mill Crk., 23.vii.1964, near stream under rocks, M.E. Irwin (1 ♂, 4 ♀, LACM), same as previous, vii.28.1965, P. Rauch (1 ♀, LACM), Idyllwild, 7.vii.1940, ALM (10 ♂, 16 ♀, USNM), Strawberry Crk., 3000', San Jacinto Mts., 19.v.1966, C.L. Hogue (3 ♂, 2 ♀, LACM), San Jacinto Peak, 7,000-10,000 ft, 20.viii.1914, J.C. Bradley (1 ♀, CUIC), Idyllwild,13.vii.1940, Timberlake (2 ♀, UCR), San Jacinto Mts, 21.vii.1929, P.W. Oman (1 ♂, EMUS), Cottonwood Cyn, 7.iii.1967, S. Frommer, H. Nakakihara, T. Plichta (2 ♀, UCR), San Jacinto Mts, 10 road mi. from Hwy 74 toward Pine Cove, Jct N Fork San Jacinto R, 4.vi.1978, S. Frommer & W. Kramer (2 ♀, UCR), Agua Caliente Indian Res., Palm Cyn, 25.ii.1970, PHA (2 ♀, CAS); San Bernardino Co., Camp O-onga, nr. Running Spgs, San Bdno Mts, 11.viii.1966, C.L. Hogue (11 ♂, 7 ♀, LACM), Mt Home Cyn, 16.viii.1920, F.R. Cole (2 ♂, 9 ♀, CMNH, EMEC), same as previous, 16.vii.1921 (1 ♀, EMEC), same as previous, 20.ix.1922 (3 ♀, EMEC), same as previous, 16.vii.1922 (1 ♀, EMEC), Mill Crk Cyn, 17.vii.1922 (1 ♂, FSCA), Mt San Gorgonio, Vivian Crk, 6500', 2.vii.1968, Truxal (1 ♂, LACM), Vivian Crk., 6500', S. Gorgonio, 2.vii.1968, C.L. Hogue (1 ♀, LACM), Forest Home, 29.v.1972, G.R. Ballmer (1 ♀, UCR), Bear Valley, San Bernardino Mts, 6700 ft, JMA (1 ♂, USNM), same as previous, N. Banks (1 ♂, 1 ♀, MCZ), Glen Martin, 16.viii.1920, F.R. Cole (1 ♀, EMEC); San Mateo Co., 2 mi. N Pebble Beach, intertidal zone, 8.v.1965, A.J. Slater (1 ♀, EMEC), Corte de Madera Crk near Portola, 22.viii.1957, PHA (1 ♀, CAS); Santa Barbara Co., Cueva Valdez, Santa Cruz Is., 31.viii.1980, RLH (3 ♀, MTEC), Fryes Harbor, Santa Cruz Is., 1.ix.1980, RLH (1 ♀, MTEC); Santa Clara Co., Uvas Cyn County Park, 2.vii.1971, D.G. Denning (10 ♂, 2 ♀, UCDC), same as previous, 9.iii.1973 (2 ♂, 4 ♀, UCDC), same as previous, 15.vi.1975 (1 ♂, 1 ♀, UCDC), Alum Rock Park, 3.xi.1975, S. Fend (1 ♀, CAS), same as previous, 21.iv.1951, R.L. Usinger (2 ♂, EMEC), same as previous, 22.vii.1979, rocks over creek, L.G. Bezark (14 ♂, 18 ♀, CSCA), Stevens Crk, 23.vii.1940, T.G.H. Aitken (1 ♀, CAS); Santa Cruz Co., Santa Cruz, 8.vii.1948, W.W. Wirth (1 ♂, EMEC); Shasta Co., McArthur Burney Falls Memorial SP, 2900', 1.viii.1970, PHA (1 ♀, CAS); Sierra Co., Hwy 49, Crk at Rosassco Ravine, 2.4 km W Downieville, 890m, 4.vii.1975, PHA (1 ♂, CAS); Siskiyou Co., S Fork Sacramento R, 4000 ft, 4.viii.1953, H.P. Chandler (4 ♂, 1 ♀, CAS); Sonoma Co., Kenwood, Sonoma Crk, Morton’s Warm Sprs, ca. 105m, 20.ix.1975, PHA (1 ♀, CAS); Stanislaus Co., Del Puerto Cyn, 900-1200', 6.vi.1970, E.I. Schlinger (1 ♀, EMEC); Tehama Co. Deer Crk, Hwy 32, 30.vii.1972, D.G. Denning (2 ♂, 3 ♀, UCDC), S Fork Battle Crk, 23.v.1952, H.P. Chandler (1 ♂, CAS); Trinity Co., 4 mi. W Forest Glen, 3400', 15.viii.1985, RLH (1 ♀, MTEC); Tuolumne Co., Pinecrest, 2-3.ix.1947, PHA (2 ♂, CNC), same as previous, 9.vii.1947 (1 ♀, CAS), same as previous, 19.vii.1948 (1 ♂, 1 ♀, CNC, FSCA), Lyon’s Dam, 8.vii.1937, T.G.H. Aitken (1 ♂, CAS), Brightman Flat, 18.ix.1974, DDW (1 ♂, 1 ♀, CAS), Lions Dam, 7.viii.1937, M.A. Cazier (1 ♂, AMNH); Tulare Co., Mineral King, 30.vii.1935 (2 ♂, 1 ♀, LACM, UCDC), same as previous, 13.viii.1956, Simonds (3 ♂, 3 ♀, CSCA), same as previous, 1964, W.E. Simonds (1 ♀, CSCA), Sequoia NP, Giant Forest, 22.viii.1917, R.C. Shannon (2 ♀, CUIC), California Hot Springs, 20.vii.1994 (1 ♂, CSCA). **Idaho:** Adams Co., Little Salmon R., 18 mi. N New Meadows, 8.viii.1979, RLH (1 ♂, 1 ♀, MTEC); Boundary Co., 5 mi SW Bonners Ferry, 16.ix.1969, W.F. Barr (1 ♀, WFBM); Idaho Co., Holly Crk, Hwy 12, 33 mi. NE Lowell, 2600 ft, 19.viii.1969, E.M. Fisher (1 ♀, CSCA), Lightning Crk, 3 mi. N Riggins, 25.iii.1966, W.F. Barr (7 ♀, WFBM), same as previous, S.D. Smith (1 ♀, WFBM), Whitebird, 3.vii.1907, JMA (2 ♂, 1 ♀, USNM), Fiddle Crk, 5 mi. N Riggins, 25.iii.1966, W.F. Barr (1 ♀, WFBM); Kootenai Co., Beauty Crk, near Lake Cd’A., 15.ix.1975, D.F. Veirs (1 ♂, WFBM); Latah Co., Moscow Mt (5 ♀, WSU), Juliaetta (2 ♀, CNC, USNM), Juliaetta, 12.viii.1904 (1 ♂, USNM), Juliaetta, 12.vii.1904, JMA (1 ♂, 3 ♀, USNM), Kendrick, JMA (2 ♂, 1 ♀, USNM), same as previous, 28.iii.1902 (4 ♀, USNM), Mtn Moscow, 25.vii.1920, R.C. Shannon (2 ♂, 5 ♀, CNC, CUIC, WSU), Lower Sand Crk, nr. Bonami Crk, 16 mi. E Potlatch, 2900 ft, 9.viii.1979, WJT (2 ♂,WSU), same as previous, 5.viii.1979 (1 ♂, WSU), Big Meadow Recreation Area, 7 mi. NNE Troy, 3000 ft, 7.viii.1986, WJT (3 ♂, WSU), same as previous, 31.vii.1979 (1 ♂, WSU), 4.1 mi. S Juliaetta, 14.iii.1975, D.F. Veirs (4 ♀, WFBM), Moscow Mt, 4.vi.1910, JMA (1 ♀, USNM), 4.1 mi. S Juliaetta, 14.iii.1975, D.F. Veirs (6 ♀, WFBM); Lemhi Co., 5 mi. N Gibbonsville, 21.vii.1963, W.F. Barr (1 ♂, WFBM), same as previous, 1.ix.1967 (1 ♀, WFBM); Lewis Co., Five Mile Crk at Clearwater R., 14.vii.1992, RLH (1 ♂, 3 ♀, MTEC); Nez Perce Co., Lewiston Hill, 25.vi.1923, ALM (4 ♂, 2 ♀, USNM), Lake Waha, 9.vi.1918, ALM (2 ♂, 1 ♀, USNM), Juliaetta Falls, 11.iv.1975, D.F. Veirs (3 ♀, WFBM), Lewiston, 9.vi.1923, ALM (6 ♂, 2 ♀, USNM); Twin Falls Co., 20 mi. S Hansen, 27.vii.1973, RLH (6 ♂, 8 ♀, MTEC). **Montana:** Cascade Co., Memorial Falls, 2 mi. SE Neihart, 6150 ft, N46°54.78', W110°41.63', 12.ix.2009, JBR (3 ♂, 3 ♀, MTEC); Flathead Co., Coram, Badrock Cyn, Shepard Memorial Fountain, swept from rocks and vegetation of seepage, 28.viii.1981, PHA (2 ♀, CAS); Gallatin Co., Silken Skein Falls, 3 mi. S. of Hyalite Rsvr., 8333', 11.viii.2000, JBR (1 ♂, MTEC), Palisade Falls, 1 mi. S. Hyalite Rsvr., 7685', 12.viii.2000, RLH & JBR (1 ♂, 2 ♀, MTEC), same as previous, 19.x.2000, JBR (1 ♂, 2 ♀, MTEC), same as previous, 20.iv.2002 (1 ♀, MTEC), same as previous, 20.viii.2009 (1 ♂, MTEC); Glacier Co., Glacier NP, Sun Point Trail, Crk below Baring Falls, 26.viii.1981, PHA (1 ♀, CAS); Park Co., Pine Crk Falls, 15 mi. S Livingtson, 6000', 6.viii.2000, RLH & JBR (3 ♂, 7 ♀, MTEC), same as previous, 28.vii.2001, JBR (2 ♂, 1 ♀, MTEC). **Nevada:** Elko Co., Ruby Mts, Thomas Cyn, 7500', 15.viii.1989, RLH (6 ♂, MTEC), Ruby Mts, Lamoille Crk, 8900', 18.viii.1989, RLH (4 ♂, 3 ♀, MTEC), same as previous, 25 mi. SE Elko, 8800 ft, 11.viii.2005, JBR & RLH (3 ♂, 2 ♀, MTEC). **Oregon:** Mt Hood, 29.vii.1966, FCH (5 ♂, 8 ♀, CAS, FSCA, MTEC, EMUS); Benton Co., Parker Crk., Mary’s Peak Rd, 26.vi.1985, R.W. Baumann, C.R. Nelson & M.F. Whiting (1 ♀, BYU), Siuslaw Nat. For., Mary’s Peak, Parkers Crk. & falls, 14.vii.1989, B.J. Sinclair (2 ♂, CNC), same as previous, Alder Falls (2 ♂, CNC), Marys Peak, Parker Crk., 3100', 24.vii.1969, E.M. Fisher (2 ♂, 2 ♀, CSCA), Mary’s Peak, Parker Crk., 26.ix.1967, KJG (1 ♂, 3 ♀, MTEC, OSAC), Mary’s Peak, 19.iii.1968, KJG (1 ♀, OSAC), Marys Peak, 14 mi., 20.v.1979, K. West (5 ♀, OSAC), Mary’s Peak, Parker Crk Falls, 3500', 27.ii.1964, J.D. Lattin (1 ♀, OSAC), Marys Peak, 11.viii.1953, V. Roth (1 ♀,OSAC), same as previous, 21.viii.1952 (1 ♀, OSAC), Cary’s Grove, 2.ix.1974, W.N. Mathis (2 ♂, OSAC), Mary’s Peak, 3.x.1978, G.L. Parsons (1 ♂, OSAC), Mary’s Peak, Parker Crk, roadside seepage, 26.ix.1967, KJG (1 ♂, CAS); Clackamas Co., Still Crk., Mt. Hood, Timberline Lodge Rd., 27.vi.1985, C.R. Nelson (1 ♂, BYU), Eagle Crk, 15.vi.1925, ALM (1 ♀, USNM), same as previous, 2.viii.1921 (2 ♀, USNM); Clatsop Co., Saddle Mt, 2500-3000', 2.ix.1966, KJG (1 ♂, ODAC); Hood River Co., Starvation Crk. St. Pk., cascading stream below falls, 10.vii.1989, B.J. Sinclair (1 ♀, CNC), Cloud Cap Inn, Mt Hood, 11.vii.1932, JMA (2 ♂, 1 ♀, USNM), Homestead Inn, Mt Hood, 12.vii.1932, JMA (1 ♂, 5 ♀, USNM), Hood River, seepage over road cut, 15.viii.1966, KJG (1 ♀, FSCA), 20 mi. S Hood River, seepage vertical roadcut, 16.viii.1966, KJG (1 ♀, OSAC); Jackson Co., 10 mi. S Ruch, stream margins, 22.v.1964, KJG (1 ♀, ODAC); Lake Co., Deep Crk, 1.5 mi. W Adel, 4850', 17.viii.1992, RLH (2 ♀, MTEC), Ana Sprs Reservoir, sweeping margin of springs, 3.viii.1966, KJG (1 ♀, ODAC) St. Helena Crk, stream margin, 17.viii.1948, W.W. Wirth (1 ♀, USNM); Linn Co., 1 mi. S Marion Forks, shaded vertical seepage, 24.viii.1967, KJG (2 ♂, 2 ♀, ODAC); Multnomah Co., Troutdale, 1.viii.1965, FCH (5 ♂, 4 ♀, EMUS, MTEC, ODAC); Tillamook Co., Neskowin, 10-19.viii.1948, M.T. James (3 ♂, 3 ♀, WSU), 4 mi. W of summit of Coast Range, Hwy 6, small crk flowing down face of road cut, 29.vii.1966, KJG (1 ♂, CAS); Umatilla Co., Dead Man Pass, 30.vii.1966, FCH (1 ♂, 3 ♀, FSCA); Union Co., North Powder, 25.vii.1965, FCH (6 ♂, 5 ♀, CAS, FSCA, MTEC, USNM), 4 mi. W Elgin, bank of Crk, vertical rock, 10.viii.1967, KJG (2 ♂, 2 ♀, ODAC, OSAC); Wallowa Co., 39 mi. N Enterprise, Hwy 3, 3400 ft, seep area, 28.vi.1976, WJT (8 ♀, WSU), 39 mi. N Enterprise, Hwy 3, 3400 ft, seep area, 28.vi.1976, WJT (1 ♂, WSU), 9 mi. S Imnaha, 1.vii.1969, KJG (2 ♀, ODAC, OSAC), 10 mi. N Imnaha, waterfall, 1.vii.1969, KJG (2 ♂, 1 ♀, ODAC); Wasco Co., The Dalles, 27.vii.1965, FCH (3 ♀, CAS, EMUS, MTEC). **Washington:** E. Washington (1 ♀, USNM); Mt Adams, 24.vii.1921, ALM (1 ♂, USNM); Asotin Co., Fields Spring S.P., 4 mi. S Anatone, 3500-4000 ft, 12-13.vi.1974, WJT (2 ♀, WSU), same as previous, 31.v.1975 (1 ♀, WSU), same as previous, 3600 ft, 26.vi.1979 (1 ♂,1 ♀, WSU), 17 mi. S Anatone, nr. Grande Ronde River, 1950 ft, dripping spring, 31.v.1976, WJT (1 ♀, WSU), same as previous, 11.vi.1976 (3 ♂, 1 ♀, WSU), Clarkston, 8.vi.1923, V. Argo (1 ♂, USNM), Asotin, 19–20.v.1923, V. Argo (4 ♀, USNM), Asotin, 4.vi.1930, JMA (2 ♀, USNM); Columbia Co., Tucanon RS, Blue Mts, 13.viii.1922, V. Argo (1 ♀, USNM); Lewis Co., Stevens Crk at Stevens Cyn Rd, Mt Rainier NP, 4000-4500 ft, 24.viii.1973, WJT (1 ♂, WSU); Pierce Co., Mt Rainier, 31.vii.1966, FCH (5 ♂, 4 ♀, CAS, FSCA, MTEC, EMUS), Mt Rainier, Christine Falls, 16.viii.1917, ALM (2 ♂, CNC, USNM), Mt Rainier, Summerland, 29.viii.1934, ALM (1 ♀, USNM); San Juan Co., Friday Harbor, 23.vii.1905, JMA (1 ♂, 2 ♀, USNM), Olga, 15.vii.1909, JMA (1 ♀, USNM); Skamania Co., Stevenson, 20.vii.1906 (1 ♀, USNM); Whitman Co., Pullman, 2100 ft, 28.viii.1984, WJT (1 ♂, WSU), Rock Lake (1 ♀, USNM); Yakima Co., Bear Crk, 8 mi. SW Tieton RS, nr Rimrock Lk. 3000 ft, 24-25.vi.1974, malaise/CO_2_, WJT (1 ♀, WSU).

### Liancalus sonorus

Taxon classificationAnimaliaDipteraDolichopodidae

Runyon & Hurley sp. n.

http://zoobank.org/82101701-D65F-4434-BD93-6A7B51F16720

Figs 2B , 3E , 5B , 10B , 13 , 16F

#### Diagnosis.

Males and females are most similar to *Liancalus genualis*, but can be distinguished by having 2 intra-alar setae, whereas *Liancalus genualis* only has 1 intra-alar seta. Males are further distinguished by having tarsus I with tarsomere 2 very short (Fig. 3E), cerci long (Fig. 2B), and wing as in Fig. 5B.

#### Description.

**Male.** Body length 7.5–8.25 mm, wing length 6.5–7.0 mm. **Head:** Face nearly parallel-sided above frontoclypeal suture, slightly widening below suture; with dense silver-gray pollen along eyes that is otherwise sparse revealing violet and green-blue reflections. Frontoclypeal suture near mid-face, distinctly bulging. Eyes with minute hairs between facets; ommatidia the same size throughout. Vertex with dense silver-gray pollen along eyes that is sparser medially revealing violet, green-blue, and coppery reflections. Ocellar tubercle prominent with 2 large setae; vertical setae two-thirds size of ocellar setae, on a small elevation; 2 postocellar setae similar in size to vertical setae; postocular setae half the length of vertical setae with approximately dorsal one-half black (approximately 12 black setae), remainder white and more slender and slightly longer. Ventral postcranial hairs (beard) wholly white. Palpus black, with rather dense silver pollen and black setae that are most dense basally, with brown pollen around insertion of these basal setae. Antenna black, first flagellomere a little longer than wide, rounded apically, arista inserted just before midpoint of dorsal edge.

**Thorax:** Scutum with bronze ground color that is mostly obscured by blue-green-violet stripes along acrostichal setae, dorsocentral setae, and around postalar area; stripe along acrostichal setae pale green and narrower than stripes along dorsocentral setae; posterior slope of scutum with two large lateral blue-green-violet spots; notopleuron and postpronotum (humerus) covered with dense silver pollen, humerus usually with some violet-green reflections; 6 dorsocentral setae; 1–13 acrostichal setae in a single row; 2 notopleural setae; postpronotum with 1–2 strong setae and often a few smaller hairs or setae; 2 presutural intra-alar setae; 1 presutural and 2 postsutural supra-alar setae; 1 postalar seta; scutellum dark metallic bronze with 6 (rarely 7) large marginal setae (3 per side), no additional hairs; proepisternum with 1 dorsal and 1 ventral tuft of white hairs. Pleura metallic green-bronze, covered with dense silver-gray pollen, without setae or hairs.

**Legs:** Coxae concolorous with pleura; remainder of legs dark metallic green-bronze, dusted with silver pollen; femoral ‘knees’ narrowly orange. Coxa I uniformly covered with white, slender hairs on anterior surface (length of hairs subequal to width of coxa I), with a few black, slender setae at apex. Coxa II with scattered white hairs anteriorly (those at apex longer and stouter), a couple black setae at apex, and a black *ad* seta just beyond 1/2. Coxa III with a black dorsal seta near 1/2. Femur II with row of short (≤ width of femur) posterior to *pv* setae on distal half, those near middle of femur white, longest and becoming black and shorter apically. Tarsus I(2) short (length subequal to width), slightly thickened, with ventral brush of setulae (Fig. 3E). Ratios of tibia:tarsomeres for leg I: 18-8-2-4-3-2; for leg II: 26-23-9-4-2-2; for leg III: 33-16-15-4-2-2.

**Wing** (Fig. 5B): Hyaline, with anterior third somewhat brownish and a diffuse brown spot near 2/3 between R_4+5_ and M_1_; with a longitudinal spurious vein between R_4+5_ and M_1_ that is arched on apical third of wing and terminates near midpoint of a nearly circular, translucent, apical spot that is white in certain lights; this spot enclosed within a small, brown, apical cloud. Calypter yellow with a fan of long, pale yellow setae at apex. Halter pale yellow.

**Abdomen:** Cylindrical, elongate (Fig. 2B), bronze with large metallic blue-green spots with dense silver pollen laterally at base of T1-T4 which do not meet dorsally (except occasionally on T1 which is largely blue-green). T5 metallic green dorsally with sparse silver pollen. T6 wholly metallic green with sparse silver pollen. T1-T3 and base of T4 with white hair laterally, longest on T1 and T2. Sternites bronze with silver-gray pollen. S1 bare except for lateral tuft of 3–5 white hairs at extreme base. S2 and S3 with sparse white hairs. S4 mostly bare. T5 with a few white hairs ventrally and a row of black setae along posterior margin. Hypopygium (Fig. 10B): cerci almost as long as abdomen, slender, cylindrical (though often shriveled when dried), sparsely covered with whorls of long, pale yellow setae.

**Female.** Body length 6.5–7.0 mm, wing length 6.5–7.0 mm. Similar to male except for face wider; palpus slightly larger and more uniformly covered with black setae. Tarsus I(2) normal, not unusually short or thickened. Wing as in Fig. 16F.

#### Etymology.

This species is named for its known distribution: most specimens were collected in the Sky Island mountain ranges in the Sonoran Desert region (Fig. 13).

#### Remarks.

Specimens were collected in April-June prior to onset of the summer monsoon.

Robinson (1970b: 57) reports *Liancalus genualis* from Mexico (Guerrero) but this single specimen "differs from the eastern North America material by…the presence of a row of small acrostichal setae". This specimen could not be located, but is probably *Liancalus sonorus*.

#### Distribution.

*Liancalus sonorus* is known from the southwestern U.S. and neighboring Mexico (Fig. 13).

#### Type material.

**HOLOTYPE** ♂, labeled: "ARIZONA: Cochise Co./ Ramsey Canyon/ Huachuca Mtns. 5500'/ 23-IV-2002/ R. Hurley & J. Runyon"; "HOLOTYPE/ ♂ *Liancalus*/ *sonorus*/ Runyon & Hurley" [red label] (MCZ). **PARATYPES:** **MEXICO:** CHIHUAHUA: Radiola Spring, tributary Rio Chuhuichupa, 23.vi.1987, Baumann, Kondratieff, Sargent &Wells (1 ♀, BYU). **USA:** same data as holotype (3 ♀, MTEC); Pima Co., Catalina Mts, Marshall Gulch near Summerhaven, 28.v.1986, J. Jenkins (1 ♂, 2 ♀, MTEC); Santa Cruz Co., Coronado National Forest, Santa Rita Mts, Madera Cyn, 3-4.vi.1991, B.J. Sinclair (1 ♂, 2 ♀, CNC), same as previous, 27.iv.1979, K.N. Barber (1 ♂, DEBU), same as previous, 5100 ft, 25.iv.2001, RLH & JBR (1 ♂, MTEC).

## Supplementary Material

XML Treatment for Liancalus

XML Treatment for Liancalus genualis

XML Treatment for Liancalus hydrophilus

XML Treatment for Liancalus limbatus

XML Treatment for Liancalus pterodactyl

XML Treatment for Liancalus querulus

XML Treatment for Liancalus similis

XML Treatment for Liancalus sonorus

## Supplementary materials

Supplementary material 1 Supplemental Video 1 Data type: MPEG video file Explanation note: Adult female *Liancalus similis* feeding on nematocerous dipteran larva (Tipulidae?) on Palisade Falls, Gallatin County, Montana on June 27, 2013. This dataset is made available under the Open Database License (http://opendatacommons.org/licenses/odbl/1.0/). The Open Database License (ODbL) is a license agreement intended to allow users to freely share, modify, and use this Dataset while maintaining this same freedom for others, provided that the original source and author(s) are credited.Justin B. Runyon, Richard L. Hurley

Supplementary material 2 Supplemental Video 2 Data type: MPEG video file Explanation note: Two adult males of *Liancalus similis* on Palisade Falls, Gallatin County, Montana on October 12, 2012. This video was taken looking upwards on vertical basalt column near water of falls. This dataset is made available under the Open Database License (http://opendatacommons.org/licenses/odbl/1.0/). The Open Database License (ODbL) is a license agreement intended to allow users to freely share, modify, and use this Dataset while maintaining this same freedom for others, provided that the original source and author(s) are credited.Justin B. Runyon, Richard L. Hurley

Supplementary material 3 Supplemental Video 3 Data type: MPEG video file Explanation note: Two males of Liancalus similis showing the typical looping flight of *Liancalus* adults. This video was taken looking upwards on vertical basalt column on Palisade Falls, Gallatin County, Montana on October 12, 2012. This dataset is made available under the Open Database License (http://opendatacommons.org/licenses/odbl/1.0/). The Open Database License (ODbL) is a license agreement intended to allow users to freely share, modify, and use this Dataset while maintaining this same freedom for others, provided that the original source and author(s) are credited.Justin B. Runyon, Richard L. Hurley
